# Citizens' views on prices of medicines reimbursed by the National Health Service: Findings from Italian online focus groups

**DOI:** 10.1111/hex.14005

**Published:** 2024-03-03

**Authors:** Cinzia Colombo, Daniele Caldara, Rita Banzi

**Affiliations:** ^1^ Laboratory of Medical Research on Consumer Involvement, Department of Medical Epidemiology Istituto di Ricerche Farmacologiche Mario Negri IRCCS Milano Italy; ^2^ Altroconsumo—Independent Consumer Organization Health Department Milan Italy; ^3^ Center for Health Regulatory Policies Istituto di Ricerche Farmacologiche Mario Negri IRCCS Milano Italy

**Keywords:** citizen, focus groups, medicines' access, medicines' price, National Health Service, public health

## Abstract

**Introduction:**

Access to medicines is one of the biggest challenges to health systems, affecting society and individuals. This study aims to explore citizens' opinions, perceptions and attitudes on the model of medicines' research and development (R&D) and price setting of medicines reimbursed by the Italian National Health Service.

**Materials and Methods:**

We run four online focus groups, analysed through thematic analysis. Inclusion criteria: people aged 30–70 years, who had completed at least compulsory schooling (8–10 years), with no specialised knowledge about the subject. Exclusion criteria: healthcare workers, pharmaceutical and device industry employees, researchers and medicine policy board members. We aimed to include a purposive sample of 20 participants, variable in terms of age, educational level and place of residence.

**Results:**

Eleven women and six men participated. The mean age was 53 years (range: 28–73). Most (*n* = 15) had a university degree or attended secondary schools. Eight had a job, five were not employed, and four were retired. In general, participants supported the role of the public health service. Almost all had limited knowledge of medicines' R&D and price setting. Most asked for transparency on medicine prices and negotiation criteria. Participants considered revenues of pharmaceutical companies disproportionate and most called for containment measures of profits. Most were in favour of a stronger public intervention in R&D and prices' negotiations. Few were sceptical of the public sector's ability to play this role.

**Discussion:**

Medicines' prices were discussed as a health matter. Increasing citizens' awareness of these topics is needed by providing spaces and conditions to participate in the discussion, including different perspectives and interests.

**Patient or Public Contribution:**

Members of BEUC—the European consumer organisation—proposed the project. Altroconsumo, an independent consumer organisation and OCU, a Spanish consumer organisation, participated in developing the project and the main topics to discuss. The Mario Negri Institute and Aplica cooperative—the Spanish methodological team—were involved by BEUC and their national organisations to define the methodology, organisational aspects and contents and conducted the focus groups.

## 1 INTRODUCTION

Access to medicines and health products is fast becoming one of the biggest challenges to health systems, with National Health Services (NHS), regulatory agencies, pharmaceutical industries, the scientific community and patient groups involved in countless debates.^1^, ^2^, ^3^, ^4^, ^5^

High prices of new medicines are major barriers to equitable coverage, together with medicine shortages. There is a need for joint efforts to support countries in developing effective access strategies. For instance, the Expert Committee for the 2021 update of the WHO Model List of Essential Medicines called for the establishment of a working group to explore policies for contending with the high prices of medicines that are considered essential, but unaffordable in many countries.^6^

Pharmaceutical companies claim that high costs for medicine research and development (R&D) justify high prices. However, many observers, including researchers, public entities and citizen groups, criticise the high prices of medicines^7^, ^8^ also considering the large contribution of public funding in the R&D of new medicines—^9^ and call for transparency in R&D costs and medicines' prices paid by different countries.^10^, ^11^, ^12^

In addition, according to several analyses, many medicines entered the market with little, no or unknown added value.^13^, ^14^

The development of new medicines, the regulatory process, price setting and negotiations by countries are aspects of public interest that affect the whole society in terms of health rights and resources of NHSs.

Citizens have a right to access data and information on these topics and to participate in the debate on the issues raised by the lack of transparency on the costs of pharmaceutical industries and the criteria used to set medicines prices.

Initiatives by patient and advocacy groups were successful in lowering medicines' prices or obtaining the production of medicines by some countries.^15^

Experiences of public participation are available where citizens were involved in deliberative processes to decide on coverage of medicines for hepatitis C or address the affordability of cancer drugs.^16^, ^17^

To broaden public participation, BEUC, the European Consumer Organisation, with the support of its national partners—Altroconsumo (Italy) Organización de Consumidores y Usuarios (OCU, Spain), and Consumentenbond (The Netherlands)—commissioned a series of focus groups in three European countries: Italy, Spain and the Netherlands. The goal was to explore the opinions, perceptions and attitudes of citizens on the current model of medicine R&D and price‐setting mechanisms.

The BEUC report was published in 2022 and summarises the main attitudes across the countries.^18^

This article describes and discusses the experience and results of online focus groups conducted in Italy.

The context was the public Italian NHS (see Box 1 for details).

BOX 1 Italy has a national, universal public health‐care system since 1978. It considers health a fundamental individual right. Its principles are universal coverage, solidarity and equity to guarantee healthcare access to all citizens and foreign residents. National Health Service (NHS) operates at central level, through the definition of essential level of care and monitoring, as well as decentralised, with regions in charge of organising and delivering health services to the population.

The Italian Medicine Agency (AIFA) is the national public body that regulates medicines for human use, under the direction of the Ministry of Health and the supervision of the Ministry of Health and the Ministry of Economy. AIFA is responsible for authorisation and monitoring of medicines. It governs pharmaceutical expenditure by defining the eligibility for reimbursement and supply for all authorised medicinal products and negotiating the price of those covered by the NHS.

Pharmaceutical companies seeking reimbursement from the NHS should provide to AIFA the scientific documentation showing the possible added therapeutic value of the medicine, information on manufacturing, marketing, sales and reimbursement in other countries. The price negotiated with AIFA is generally valid for 24 months. Managed market entry contracts, including financially based (e.g., price‐volume) and outcome‐based agreements, are applied to innovative, high‐priced medicines.^39^

## 2 MATERIALS AND METHODS

The manuscript's reporting follows the COnsolidated criteria for REporting Qualitative research Checklist.^19^

The Italian research methodological team (IRFMN), BEUC, Altroconsumo, the Spanish methodological team (APLICA cooperative) and OCU discussed and agreed on the methodological framework, selection criteria, key topics to cover, main sources and main issues to propose for discussion.

Focus groups were chosen due to the capacity of this methodology to delve into participants' perspectives providing a nuanced understanding of complex phenomena.^20^

### 2.1 Participants and sample size

Eligible participants were people aged 30–70 years, who had completed at least compulsory schooling, that is, 8–10 years of education. This age limit was aimed to include persons likely to be interested and have familiarity with the topic. The target was both healthy people and people living with a medical condition. To facilitate sharing and discussion, people with no specialised knowledge about the subject were selected. Healthcare workers, pharmaceutical and device industry employees, researchers, or medicine policy board members were excluded.

The aim was to include a purposive sample of about 20 participants, to reach a variable sample in terms of characteristics (such as age and level of education).

### 2.2 Invitation and selection of participants

Participants' contacts were gathered—from 7 to 16 February 2022—through a network of persons reached by the authors who were asked to contact people potentially interested in participating in the focus groups. We invited interested people by e‐mail or phone to participate in a study promoted by an association of European consumers on issues related to the health service. We did not give details of the research topic so that participants could not prepare their answers in advance.

Potential participants received an information leaflet explaining the study in general and the process for data collection and processing. Those who agreed to participate signed both a written informed consent form for the study and a data processing consent form. Participants received compensation (a 40‐euro bonus) for their attendance.

The ethics committee's approval was not required for this kind of study under Italian law.

### 2.3 Information given to participants

We defined key topics related to the price of reimbursed medicines. We set up focus groups combining a plain‐language presentation and questions to collect participants' opinions and perceptions (Appendices S1A and S1B), as we assumed that the general population's knowledge of these topics was limited. Participants had the time to discuss the key topics gradually and then to address them from a practical perspective with the support of an example (sofosbuvir).

The presentation included three sections:

1. general views and experiences on medicines and health services;
2. R&D of medicines, R&D costs and confidentiality, revenues by pharmaceutical industries and public funding.^21^, ^22^, ^23^, ^24^, ^25^
3. medicine price and reimbursement procedures, the confidentiality of actual medicine prices, the case of sofosbuvir, factors at stake in defining the price—including national or local discounts—national public expenditure on medicines, added therapeutic value of a new medicine.^26^, ^27^, ^28^, ^29^

The team selected the sources to give the participants balanced information, providing data from different viewpoints (an independent medical journal, academic researchers and pharmaceutical industries). For example, data on R&D costs^22^, ^23^, ^24^ are highly variable depending on the type of calculation and assumptions and often reflect different perspectives. We chose the example of sofosbuvir as it well highlights the issues at stake. For instance, we discussed the role of the public and private sectors in R&D, R&D costs, variability of prices among countries, debate on medicine access, the impact of saving lives of persons with hepatitis C and avoiding risks and costs of liver transplant and the increase in expenditure due to medicine price.

To test the clarity of the slide set, we asked for feedback from a person not involved in the project, with no specific expertise in the field. We then changed the layout of a couple of slides to clarify the data and explained some data in more detail.

### 2.4 Data collection

Given the restrictions due to the coronavirus disease 2019 (COVID‐19) pandemic, we organised online focus groups of 4–5 participants each. One researcher experienced in facilitating small group discussions conducted the focus group (C. C., senior researcher, doctor in philosophy) and another (R. B., senior researcher, clinical pharmacologist) expert in drug regulatory policies made the presentation. Participants were free to ask questions and make comments at any time during the presentation. At the end of each explanation, participants could suggest other topics of interest. The facilitator proposed some issues for discussion.

The meetings were recorded upon consent. Both the facilitator and the other researcher took notes, and the meetings' contents were transcribed verbatim. Transcriptions were not returned to participants for correction.

### 2.5 Data analysis

We conducted a deductive/inductive analysis. As the deductive analysis, we grouped texts and excerpts according to the proposed key topics. Within those topical areas, we analysed the text following the thematic analysis indications.^30^ An inductive analysis was conducted on the other topics that emerged during the discussion.

A researcher fractured the text into labels. Then, she clustered the labels into main and subthemes. The other researcher revised the main and subthemes and checked them for internal consistency. Identified themes were then discussed with the research team. Descriptive statistics summarise information on demographics, educational level, place of residence and work conditions.

## 3 RESULTS

We run four online focus groups—three on 24 and 26 February and one on 5 March 2022—using Microsoft Teams. From a network of 27 persons with different backgrounds, working areas and places of residence, we gathered and invited 26 potential participants. Three were not eligible because they worked in the field of healthcare, three were not available on the planned dates, and three did not respond to our invitation. We finally included a group of 17 people (Table 1). Two participants were slightly outside the eligible age range, but it is unlikely that this may have biased the focus groups.

**Table 1:** Characteristics of participants.

Number of participants	17
Gender	11 Women and 6 men
Age, mean (range)	53 year (28–73)^a^
Educational level	
University degree	8
Secondary school	7
Upper primary school	2
Working condition	
Having a job	8
Not employed	5
Retired	4
Place of residence	
South of Italy	2
North of Italy	15

^a^ One missing value.

Participants did not know the researchers involved in the focus groups, and few of them had heard about IRFMN activities.

The meetings lasted around two and a half hours.

## 4 RESULTS BY THEMES

We detail below the results by main themes and subthemes (Table 2), with quotations.

**Table 2:** Overall findings grouped around main and subthemes.

Main themes	Subthemes
Favourable attitude to public health	Supporting the National Health Service (NHS).
	Variable awareness about the functions and responsibilities of the NHS.
	Considering medicines first and foremost as a health issue.
	Calling for a stronger public role with some concern from a few about the public entities' ability.
	Wondering if public spending in R&D has a return.
Calling for transparency	Asking for transparency, particularly on medicines' prices.
	Paying attention to the psychological and emotional effects on the person who has to take expensive medicines.
Different perspectives on medicines' value	Variable attitude to medicine use in daily life.
	Defining the value of new medicines has proven to be a complex issue.
Variable attitudes towards pharmaceutical companies	Considering pharmaceutical companies as purely a for‐profit enterprise.
	Acknowledging the need for pharmaceutical companies' investment in R&D.
Call for action to address high profits of pharmaceutical companies	Astonishment about the revenues by pharmaceutical companies.
	Criticising the huge revenues.
	Asking for a limit on pharmaceutical companies' profits.
Medicines' pricing is a new issue for participants	Scant knowledge of R&D and the negotiations of medicines' prices.
	Being interested but slightly frustrated.

Abbreviation: R&D, research and development.

### 4.1 Favourable attitude to public health

#### 4.1.1 Supporting the NHS

The NHS was considered important as a public service allowing citizens to access healthcare services and medicines otherwise unaffordable, paying out of pocket. Some participants raised critical aspects related to the variable quality of hospital services and the waiting lists. Many criticised the need to pay privately for faster visits to public hospitals. The faster access available through private insurance was commented positively by a participant. Few tied the lack of resources and unsatisfactory quality of hospital services to the local conditions of some regions.

Regarding the health service … it is a pearl that must be preserved, and unfortunately, it has been mistreated for many years here. I say that we need to invest more. (FG1)

…All in all our health service, compared to certain other situations abroad, is better, that's it. (FG3)

Luckily a few years ago my company took out an insurance contract that covers part of the medical checks, so we can do something privately…. Otherwise, if you have to wait for a visit with the National Health Service, you ask for a visit, they tell you eh … I'm sorry lady we don't even have a place in 18 months, and the only alternative is to go privately … when? The day after tomorrow. (FG1)

#### 4.1.2 Variable awareness about the NHS

Participants showed varying levels of awareness about the functions of the NHS in terms of the reimbursement process and accountability towards civil society.

When discussing medicines reimbursed by the NHS, several times, participants referred to medicines in general, including over‐the‐counter medicines and supplements.

#### 4.1.3 Considering medicines as a health issue

All participants considered medicines as a matter of health, although some thought that pharmaceutical companies produce medicines to legitimately make profits. Some criticised the market perspective.

We are talking about life‐saving medicines … why ‘market’? I can sell cigarettes—anyone who wants to smoke, to hurt themselves, can do it, and I can sell any other stuff, but let's find … a different word for medicines. Medicines should be distributed for free. (FG3)

#### 4.1.4 Calling for a stronger public role with some concern from a few

Most participants were in favour of stronger public intervention in R&D and negotiations of medicine prices. Almost all expressed the need to limit the pharmaceutical companies' profits in order to afford medicines' prices and the sustainability of the healthcare system.

I find it little strange that most of this work (early phases of R&D) is done by universities—therefore by the public—then investors arrive—which are pharmaceutical companies—who see which research, among the large amount done, can be productive and make profits. If we consider that pharmaceutical companies are among the most prosperous on the planet … I would say: why doesn't Italy do it? That is, why don't the states, even united, do that pivotal part (of research)? (FG2)

When dealing with the process of price setting and reimbursement, and the issue of confidentiality on the price actually paid by governments for a specific medicine, an entity monitoring the process of negotiation was required, and public tenders were suggested.

… the same medicine produced by company X is sold in Italy at a price that is set by the Italian drug agency, in short, by the government, and in France it could cost less, it could cost more (…) it does not seem to me a very correct thing … so the question is perhaps not about the medicine, but then … what is Europe for? (FG3)

It could also be useful at the state level—like for other sectors—to be able to say: company ‘X’ produces a medicine (…) with this active ingredient, and another pharmaceutical company produces the same product with the same active ingredient. These two pharmaceutical companies could bid, with the Italian state, so that the one with the lowest price goes to the regions, or hospitals. (FG4)

A few doubted the ability of public entities to play a stronger role in the R&D of medicines and negotiations (see Appendix S2).

#### 4.1.5 Wondering if public spending in R&D has a return

Some participants wondered what is the return of the public funding, in terms of transparency on costs, usefulness and quality of medicines produced (see also Appendix S2). A few commented that the public sector has delegated too much to the private one.

If public entities fund (research), do they have access to confidential information from the private pharmaceutical company or they simply pay but have no return, no feedback? (FG3)

I would be curious to know the return on the public investment, in terms of utility and quality of drugs developed using this funding. (FG4)

### 4.2 Calling for transparency

#### 4.2.1 Asking for transparency, particularly on medicines' prices

While few participants discussed R&D cost transparency, those who commented were mostly in favour of more transparency and public disclosure of costs (see Appendix S2), asking for a change in the regulations. One said that pharmaceutical companies are allowed by law to maintain confidentiality on costs for commercial reasons and, to oblige them to disclose their costs, the public should have a stronger role.

(…) if there is a state intervention to cover the costs as a national health system (…) the costs should be public, I would like to know—as a State—what I pay and what is behind it: studies, research, why I am asked that price. (FG3)

If a public entity has the obligation to disclose, in this case they are private companies that receive partial funding… (…) They are private companies, which have to finance patents, which have obligations, when they do research, then afterwards they have to protect themselves in some way … morally I would like to see everything too, but from the practical point of view I say ‘how much do we invest in research so that we can … oblige these companies to make their results public?’ (FG3)

Many participants asked for transparency on medicines' prices, including on negotiations and discount criteria. The topic was discussed broadly when dealing with the sofosbuvir example.

Two main positions emerged. One asking for transparency, underlining that public administrations should be transparent to be accountable.

Personally I do not feel comfortable about pricing being confidential, because a relationship between the private company and the public administration should be transparent. (…) The fact is that public money is used for a confidential price. I swear I did not know this and it worries me a lot. (…). (FG4)

#### 4.2.2 Paying attention to the psychological and emotional effects of disclosure

The other position—by a few participants—supported confidentiality on medicine prices towards the public to avoid people with a disease feeling guilty about their burden on the NHS and discrimination.

I honestly find it correct (confidentiality), because, as citizens, we cannot deal with a price on our health. (…) I find it extremely fair not to disclose the price. Not only from a point of view of business mechanisms, (…) but also at the level of conscience. (…). I would leave this arduous task to those in charge (…). (FG4)

### 4.3 Different perspectives on medicines' value

#### 4.3.1 Variable attitude to medicine use in daily life

Most participants used medicines to treat chronic conditions or diseases, such as hypertension, hypercholesterolaemia, cancer and panic attacks. The general attitude towards medicines' use tended to vary. Some used medicines only when strictly necessary, while others expressed a broader attitude (see also Appendix S2).

Well … clearly I'm 35, 36 … and so, for now, I haven't much needed (medicines) … Even when it happens … I try to … to put it off if I can, in the sense that I try not to take medicines at all. (FG2)

(…) Drugs, at least as far as I am concerned, for ordinary health problems, are a blessing for me. (FG3)

#### 4.3.2 Defining the value of new medicines is a complex issue

We presented data from an independent analysis^21^ discussing the number of medicines that were considerably better than existing treatments, those providing little advance, those bringing nothing new or were worse and those lacking data to know if they bring value to patients.

Many asked for clarification, in particular, if the medicines of ‘unknown value for patients’ included those with the same indications as medicines already available but with better safety profiles, or generic medicines or supplements.

The most frequent reaction was surprise. Some participants were used to thinking that new medicine means better medicine (see Appendix S2), so the small number of new medicines proved to be advantageous for patients surprised them. Some others were almost sorry or disappointed, a few were not surprised at all. They underlined that much research is done for economic reasons and that the rush towards so‐called innovation is a ‘trick’ to sell medicines for profits.

Honestly, I was a bit perplexed, because I thought that research always tended to develop … eh … medicines that were more advantageous than the previous ones. So I did not imagine, honestly I am really very surprised, negatively (…) also maybe the laws should change, because if there is so much effort in research, it would be more appropriate … to strive to improve existing things, not create other things. (FG3)

I'm honestly not surprised. (…) I think that a lot of pharmaceutical research is essentially driven by the economic discourse, so it is very easy to do research like this, to offer ‘novelties’, also because we are all very attracted to ‘novelty’ and I think that the trick is a little bit that, right? (FG1)

When discussing the value of a medicine, different positions emerged. One stated that it is a complex issue that brings together economic and ethical aspects. Another criticised the use of the word ‘value’ for a medicine, referring to the economic value. Two other positions argued the impossibility of distinguishing among medicines in terms of value: the first because a medicine has the highest value when a person needs it; the second because there are no ‘first’ and ‘second class’ medicines. A further position considered that the value of a medicine depends on the severity of the disease.

…an ethical value of something that saves a person's life, (.) it is hard to give it a value. I think any of us would pay any sum to have a medicine that saves the life of someone. The ethical part … it always has to be there, so (…). If the value is purely economic, (…) the various costs … a lot of money. For me, whose life has been saved (…). I accept these unfair conditions. For us who look at it from outside, I understand that this value of a medicine must have an ethical and also an economic part and the economic part cannot be so exaggerated. Therefore, I don't know what the right solution is. (FG3)

In my opinion … ethics within this mechanism is not really there, meaning that if we think that health and therefore something that has no value, is placed in the hands of someone who thinks in terms of value, clearly there can be no ethics. In other words, those who do business in my opinion do business and nothing else. (FG2)

### 4.4 Variable attitude towards pharmaceutical companies

#### 4.4.1 Considering pharmaceutical companies as purely a for‐profit enterprise

One position strongly criticised the predominant role of the pharmaceutical industry, in a process of ‘business for profit’.

In my opinion, the criterion (to decide which medicine to develop) in the end is just to sell, and that's it … let's say the pathologies that are spread globally, while for rare diseases (…), investments are laughable and some drugs are even no longer produced. (FG2)

Profits and marketing purposes of the pharmaceutical companies were stressed by participants, also in terms of pushing on the market for new medicines with no added value.

Can pharmaceutical companies put pressure like this? To facilitate … placing the new medicine on the market and perhaps leading to the removal of an existing medicine, already tested and verified over several years? (FG3)

A participant related the R&D costs to the costs that the pharmaceutical industry is willing to invest to boost the medicine on the market, mentioning lobbying and corruption.

So this is, in theory, the cost of one medicine alone? 2 billion. (…) I imagine that we will see later how much it is worth for a company to produce a medicine … otherwise I think they would not do it. (…) Let's say I spent 2 billion euros, let's say, can I spend another 500 million euros to create a commercial network? So how much is corruption worth? How much does a politician cost? (FG2)

When discussing the different estimates of R&D costs, among the few participants comparing them, one of the main interpretations was that independent researchers are the trusted source, while pharmaceutical companies tend to exaggerate the costs to justify higher profits.

The cost is very high … I am very surprised … I knew it was high, but (…) the first estimate [highest figure] is linked to companies, every company tends to say that it has spent more … also to reduce its taxes (…) I would only take these two [lower figures], but precisely because they come from independent researchers and in my opinion, this is fundamental in this field. (FG3)

#### 4.4.2 Acknowledging the need for pharmaceutical companies' investment in R&D

The other position acknowledged that both public and private entities are needed and accepted that R&D of medicines has unavoidable economic aspects tied to the resources required, for example, the people working on it.

…It is hard for me to imagine that a medicine comes out at no cost … someone's cost there must be, right? (…) On the other hand, I personally have sometimes had to use medicines even for a long time and I was really impressed. For example, one of these medicine costs 250 euros, another … 400 euros. I think I paid roughly two euros and fifty cents for it. Personally, I cannot work out if 400 and 200 is a cost that covers ten thousand compounds that become 250, which become 5 compounds, which represent 10 years of work by those who have made an investment. (FG3)

A few participants underlined that without profits, the pharmaceutical companies would not invest in R&D. The public sector was not considered a proper alternative by a few participants because of the lack of efficiency and organisation.

It is certainly impressive to see the profits on these products. It is also true that if there were no aims of this type, who would think of investing 280–300 million dollars, if there is no objective to achieve? If this were to be done by a state, I strongly fear that a goal would never be reached because unfortunately (…) when the state is involved then, since the thing belongs to nobody, things do not lead to success. (FG2)

When the various R&D cost figures were discussed, one position, less frequent, was that researchers linked to pharmaceutical companies estimated higher costs because they had more information.

I would not discard the higher estimate … anyway they are researchers … the fact of being linked to pharmaceutical companies may have a positive aspect (…) you could know the dynamics better and therefore could have a clearer idea of the costs. (…) a voice closer to the company is able to get more information…. (FG3)

### 4.5 Call for action to address high profits of pharmaceutical companies

#### 4.5.1 Astonishment about the revenues by pharmaceutical companies

Some participants had difficulty understanding the data on costs and revenues by pharmaceutical companies presented in this section,^22^, ^23^, ^24^ requiring clarifications.

Almost all were astonished by the wide gap between revenues and R&D costs of medicines. A participant who, in the previous sections, had tried to assess the information, including the pharmaceutical company's possible perspective, did not find the data on revenues reasonable and asked to be reassured with possible explanations. Another wondered whether medicines with higher revenues were better than others, or if they were used to treat more frequent diseases. A few were not surprised.

I am shocked (…) well I understand, it is obvious that … but … this exaggerated disproportion between the cost of research and then the revenues…. (FG1)

I understand that there may be a part of the market—but I want to hope that it is not what is represented here. That there really is another reason here. Maybe someone will explain the other reason to me, so I feel more reassured…. (FG3)

These profit margins … oh well, they are quite well known, in the sense that I expected them…. (FG1)

Most commented that, given the high costs for R&D, pharmaceutical companies should expect to make high profits from medicines and marketing products that can give a good return on investment. At the same time, there was surprise over the extent of the costs.

The impact on the sustainability of the NHS was mentioned. One participant commented that if individual citizens—and not the NHS—have to bear the burden of a medicine price, a revolution would take place. She said she felt taken care of by the NHS, which reimburses the medicines she needs.

I did not expect … a service of this type to the citizen. I … who have had health problems for years, I have to say that I feel pampered and it is a beautiful form of solidarity that I would never, ever have expected. (FG4)

#### 4.5.2 Criticising the huge revenues

Participants—even those who acknowledged the need to make profits by investments—disapproved of the disproportionate revenues by pharmaceutical companies.

Discussing the example of sofosbuvir,^26^, ^27^, ^28^, ^29^ a few found it difficult to believe that the same medicine had such different prices in different countries. Most were impressed by this huge variability and by the revenues, considering the price of the generic medicine and the production cost of sofosbuvir.

Most participants made an attempt to explain the variability of prices, mentioning general market mechanisms, by which prices are set according to what each country can pay. Few commented that pharmaceutical companies offer lower prices in some countries as a sort of ‘ethical’ choice. However, almost all were critical of the high revenues.

What I see is just this: those who can pay more pay more, those who can pay less give less, but in the end everyone pays. I hope that those who pay higher prices contribute to the adequate care of those who have less financial resources…. (FG1)

On the one hand there is undoubtedly the success of having found these medicines that save lives … it is the most important thing that interests us citizens, patients. On the other hand, well, these costs are enormous … and we must bear them. (FG3)

I do not know in how many other sectors of the economy or production there are margins like those and that is quite sad because this is money on the health of citizens, and public money (…) maybe we need to review the roles of patents. (…) There should be rules, market rules also for pharmaceutical companies.…. I am completely liberal, (…) for the free market, but not (…) in certain sectors, such as the pharmaceutical one, which has an impact on the life and health of citizens and on the finances of the State. Above all, the free market, cannot exist when patents take over. (FG4)

#### 4.5.3 Asking for a limit on pharmaceutical companies' profits

Most participants commented that there is a need to balance between R&D costs and revenues by pharmaceutical companies. A few explicitly called for a ceiling price or a ceiling percentage of profits, considering they are related to the health of citizens, and called for the role of a supranational public entity in negotiations, either the European Union (EU) or the states altogether. A few suggested limiting the duration of patents (see Appendix S2).

…There will certainly be some kind of ceiling … there should be … because otherwise this is really large‐scale speculation, speculation on health. (…) It could undermine the National Health Service, which has a series of tasks to accomplish. (FG1)

I'm selling it for double the cost, and that's fine. …It is also necessary to (…) cover the research costs…. What I feel is not good is all these protected, confidential transactions that have these exaggeratedly large margins (…). If I were—I don't know, I'm not saying Italy, but all Europe, all the states united—I would begin to say ‘no, we need to change the rules’ because you can't sell me a medicine that costs 100 euros for 37 thousand euros. We are talking about lifesaving medicines, it is unacceptable. (…) we are talking about health, why are there transactions private? (…). We too could begin to think about a bit of a revolution, if we all get ourselves together. (FG1)

### 4.6 Medicines' pricing is a new issue for participants

#### 4.6.1 Scant knowledge on R&D and the negotiations of medicines' prices

Most participants did not know the price of reimbursed medicines. The majority expressed uncertainty on who usually pays for medicines' R&D. Some thought pharmaceutical companies, and others, pharmaceutical companies with the support of public entities, for example, universities. A few mentioned the example of anti‐COVID‐19 vaccines where public entities contributed to pharmaceutical companies' R&D.

A few participants said they had no idea about what drives the development of new medicines. Some guessed the criterion was the investment in the most profitable sectors; a minority suggested epidemiological factors or unmet needs. Finding medicines with less adverse effects, or extending the use of medicines from one condition to others were also mentioned (see Appendix S2).

#### 4.6.2 Being interested but slightly frustrated

At the end of the meetings, some participants expressed their intention to keep the focus on these issues, thanking that they had the opportunity to address them. Many asked for more information from the public. Some expressed a sense of frustration in relation to the huge interests at stake and the possibility of changing the rules.

## 5 DISCUSSION

To our knowledge, this is the first Italian experience of citizen focus groups on determinants of prices of medicines reimbursed by the NHS and price negotiations.

As expected, almost all participants had limited knowledge of the data and information presented. The majority had not thought about medicine prices before. In general, the views expressed by the participants supported the role of the public health service and the need for transparency on medicine prices. Participants had varying attitudes towards pharmaceutical companies, acknowledging the need for their investments but asking for ways to balance medicine prices with R&D costs and pharmaceutical company's profits. Different perspectives of the value of a new medicine emerged, due to variable understanding of the word ‘value’, linked to economic perspective, but also to the disease and its experience.

Similar findings emerged from the focus groups in the Netherlands and Spain.^18^ Participants were astonished that only a few new medicines proved to be considerably better than medicines already available. They did not approve of high drug prices and the huge amount of revenues by the pharmaceutical companies, asking for a stronger role of the government in price negotiations, with access to information on R&D costs. In a different setting, in the United States, the majority of the persons responding to a poll considered profits by pharmaceutical companies as the major factor contributing to the price of prescription medicines.^31^

In Italy, health expenditure is lower than in other Western European countries, and while three‐quarters of the total health expenditure is funded by public sources, the percentage of private health spending has increased, in the last decades.^32^ The debate on equity of care and sustainability of the NHS is growing, and the issue of medicine price is understood as affecting citizens as taxpayers and the whole society.

Activists' groups had historically a crucial role in the pharmaceutical companies obtaining lower prices, governments' actions to afford medicines' access costs^15^ and advocating for the affordability of medicines.^33^ Many patient groups often have a sound voice when a new drug comes into the market asking for access. Not entering here in the debate on the appropriateness of the requests, the occasions to dialogue on these issues should be increased.

Methods and experiences of public and patient involvement in prioritising medicines' access, both formal and informal, are available, even if some of these approaches still need to be improved.^16^, ^17^ Strategies for measuring, monitoring and managing prices are essential for promoting access and ensuring sustainability. Several policy actions can be taken at the national and European level.

The debated issue of affordability of medicines has been specifically addressed in the new pharmaceutical strategy launched by the European Commission in 2020.^34^, ^35^

New legislation should support R&D models that fulfil unmet medical needs and ensure the accessibility and affordability of medicines.

The proposed changes in the EU pharmaceutical legislation include the involvement of patient representatives in the Committee for Medicinal Products for Human Use.^36^ This could be an opportunity to strengthen the voice of patients and broaden the discussion on access to medicines at the public level. To this end, the issues of representativeness, transparency and conflict of interest need to be addressed.

Several analyses showed that new medicines seldom have little or no therapeutic added value^13^, ^14^, ^21^ and that many pharmaceutical companies invest more in administrative, marketing activities and share buybacks than in R&D.^7^ Incentives to link the demonstration of an added benefit, a true clinical innovation to the price‐setting mechanism, should be discussed by various stakeholders. Greater transparency on R&D costs and actual prices paid in the different countries is needed to sustain NHSs in serving the public. The World Health Assembly resolution on improving the transparency of markets for medicines could be a step in this direction.^12^

## 6 STRENGTH AND LIMITATIONS

The project was developed through a collaborative approach among consumer groups—BEUC, Altroconsumo and OCU—and researchers from the Mario Negri Institute and APLICA cooperative. The methodology, the criteria for selecting participants, the framework of topics and the sources of information were discussed and shared among the working group and adapted to the local context where needed.

The study's main limitation is the potential for selection bias, due to the method of contacting and inviting participants. The organising entities—IRFMN, Altroconsumo—are known to be active in promoting accessibility and universality of the Italian NHS. Persons who decided to participate might have had a similar perspective, with a favourable attitude to public health systems. While acknowledging this, we did note that few participants had heard about IRFMN activities. In addition, we noted that participants actually offered a range of opinions. Although focus groups are not meant to be representative, more women and people from the North of Italy participated.

During the focus groups, we presented reference information to engage people in the discussion, considering the complexity of the topics and the general lack of knowledge about them. We cannot exclude that the information and how it was presented might have influenced the participants. We addressed this issue by trying to balance data and statements—as reported in Section 2. Moreover, the researchers facilitated the participation of all people and the expression of different opinions.

It is possible that some information was too technical to be fully understood by nonexperts in the field.

## 7 CONCLUSIONS

This study provides an insight into Italian citizens' knowledge and opinions on the issues related to medicines prices, the process of negotiation and the role of the public and private sector in R&D. Recognising that the process and mechanisms of medicine pricing have consequences for the health of each person and society as a whole, it is essential to increase citizens' awareness of the factors at stake, providing spaces and conditions for participation in the discussion on access to medicines, reimbursement criteria and pricing.^37^, ^38^

## AUTHOR CONTRIBUTIONS

**Cinzia Colombo**: Conceptualisation; methodology; investigation; writing—original draft; formal analysis; data curation; project administration. **Daniele Caldara**: Conceptualisation; writing—review and editing; methodology. **Rita Banzi**: Conceptualisation; investigation; writing—review and editing; methodology; formal analysis.

## CONFLICT OF INTEREST STATEMENT

Cinzia Colombo and Rita Banzi have no conflicts of interest. Daniele Caldara works for Altroconsumo—Independent consumer organisation—funder of the study.

## ETHICS STATEMENT

The ethics committee's approval was not required for this kind of study under Italian law. A mandatory ethical approval is only required for clinical studies on medical products according to the Italian legislation on ethic committees ‘Definizione dei criteri per la composizione e il funzionamento dei comitati etici territoriali’ (23A00853) (GU Serie Generale n.31 del 07‐02‐2023) and the European legislation on clinical studies ‘Regulation (EU) No 536/2014 of the European Parliament and the Council on clinical trials on medicinal products for human use and repealing Directive 2001/20/EC’. This study did not involve patients, but citizens who were invited to talk about medicine prices and who signed an informed consent form and a data processing consent form (both written) after receiving information about the study. The study did not involve a medical product.

## Supporting information

Supporting information.

Supporting information.

Supporting information.

## Data Availability

Original data will be available under request, after anonymization. The full transcriptions of the focus groups contain potentially identifiable and sensitive information. They could be available on request from the author after anonymization.
