# Supplementary material for: Citizens' views on prices of medicines reimbursed by the National Health Service: Findings from Italian online focus groups
Source: Health Expect. 2024 Mar 3;27(2):e14005. doi: 10.1111/hex.14005 (PMC10909621; doi:10.1111/hex.14005)
Supplement: Supplementary file 1 — Supporting information. [file HEX-27-e14005-s001.pdf]

Appendix 1a. Slide presentation and questions.

Slides presented during the meeting and main questions asked to participants.

The questions were adapted in each meeting based on the discussion.

Opinioni e punti di vista dei cittadini su aspetti relativi al servizio sanitario nazionale e alla salute

Di cosa parleremo oggi in particolare

Il prezzo dei farmaci rimborsati dal Servizio sanitario nazionale

Parte 01

Opinioni sui farmaci e sul servizio sanitario nazionale

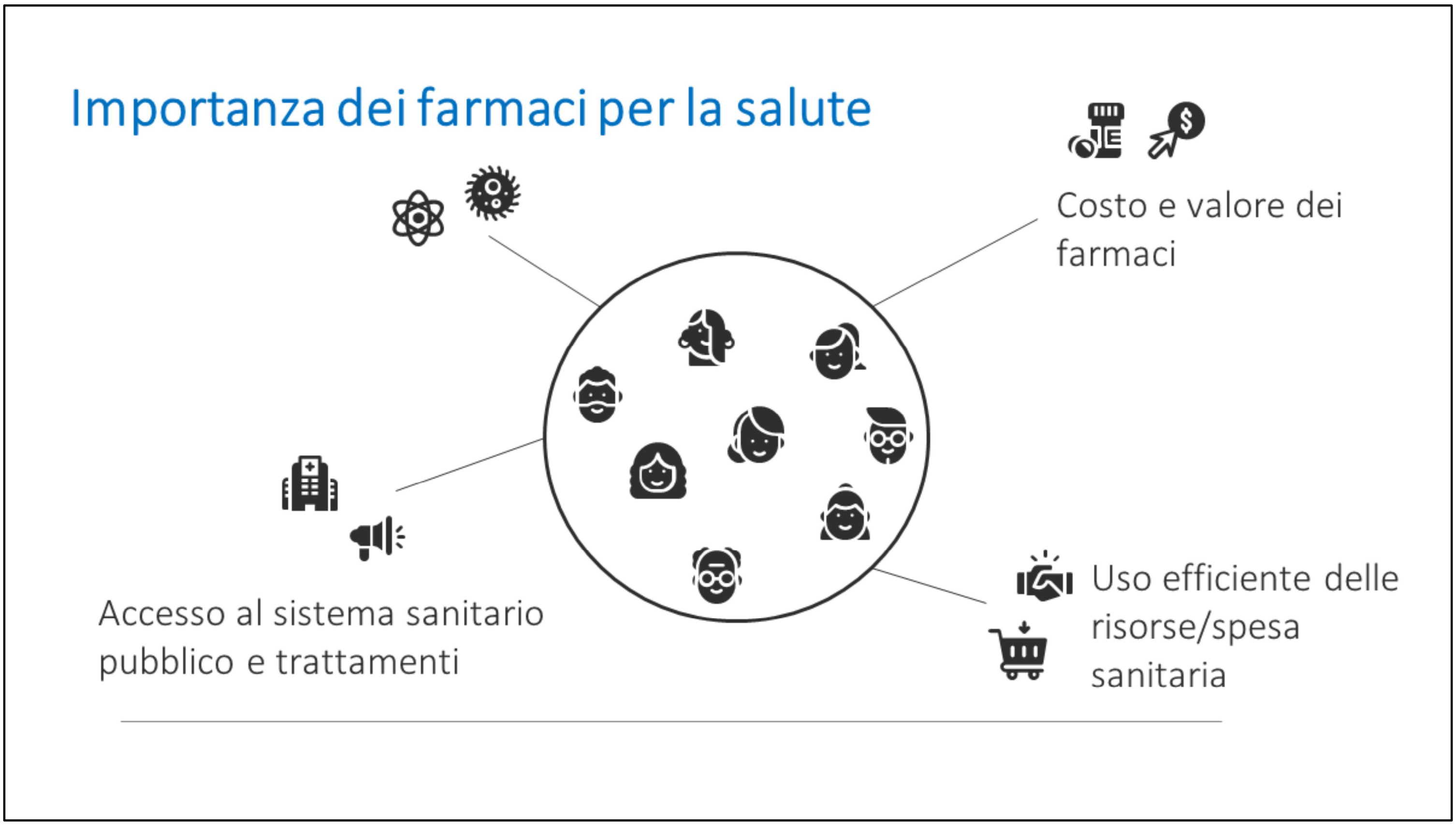

Domande

- In termini generali, che valore date alla salute?
- E all'assistenza sanitaria?
- Che valore hanno i farmaci nella vostra salute?
- Vi siete mai chiesti quale è il prezzo dei farmaci che utilizziamo che sono a carico del servizio sanitario pubblico? (per esempio i farmaci contro il diabete, o farmaci usati per prevenire la formazione di trombi come gli anticoagulanti orali)?
- Quanto costa secondo voi portare sul mercato un nuovo farmaco? (ordine di grandezza: ….)
- Siete a conoscenza di come avviene il percorso di immissione sul mercato di un nuovo farmaco?

02 Parte

Sviluppo e costo dei farmaci

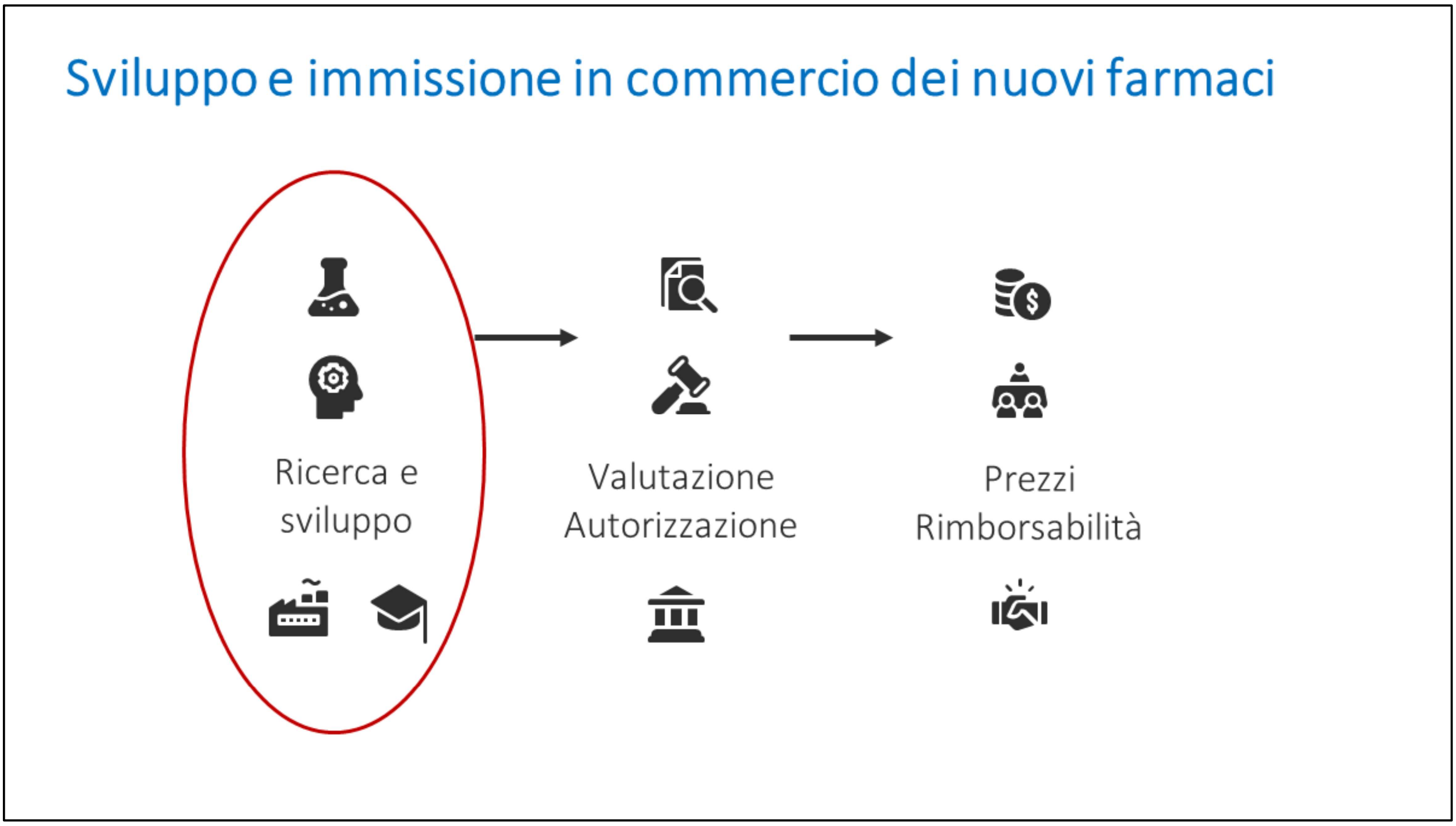

Domanda:

Secondo voi/a vostra conoscenza, chi finanzia la ricerca e lo sviluppo di nuovi farmaci?

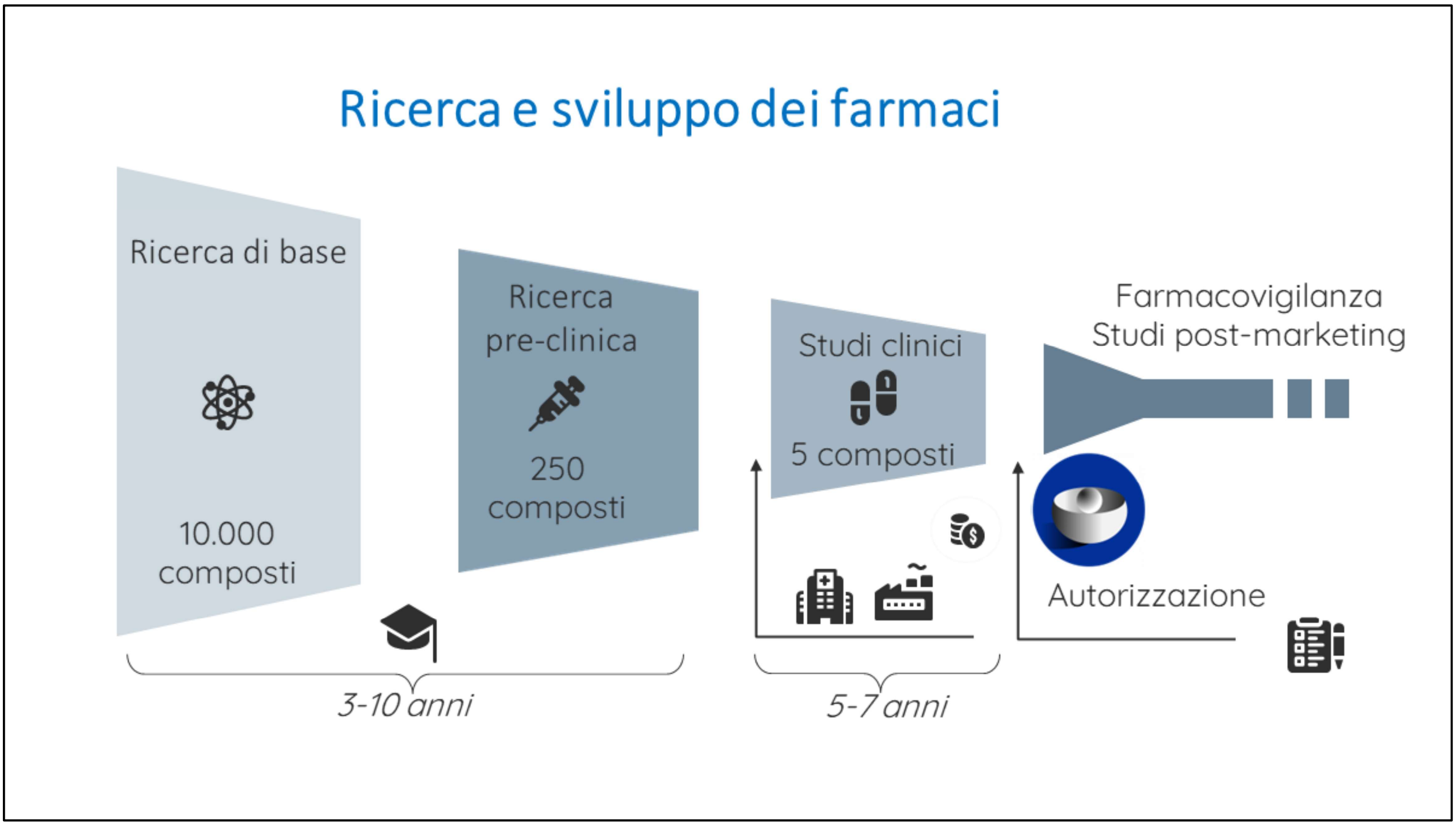

Domande:

- Avete domande? Commenti? Vi sembra tutto chiaro?
- Secondo voi, perché/in base a cosa si decide di studiare, sviluppare un nuovo farmaco…?

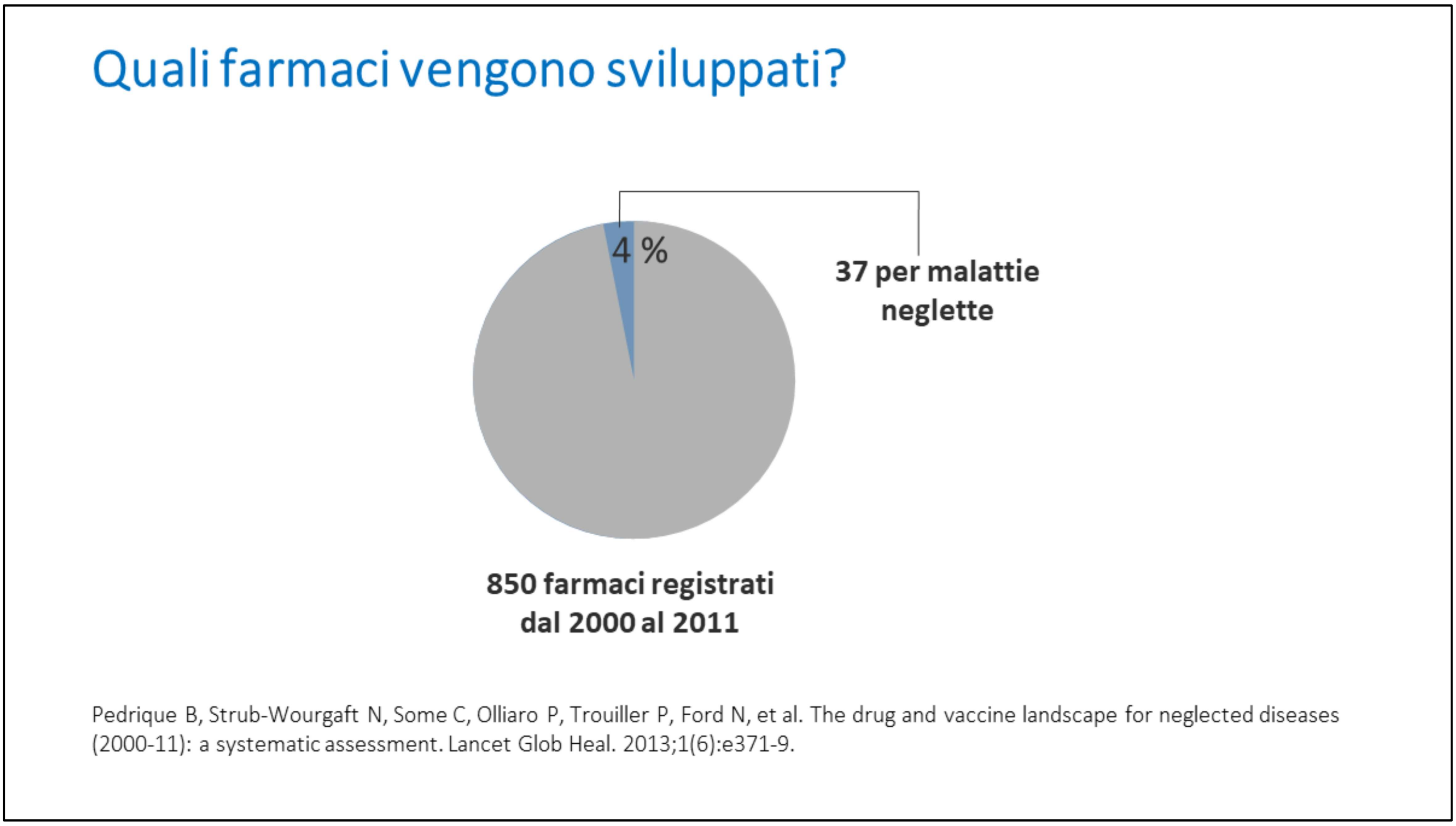

Domande:
Avete domande? Commenti? Vi sembra tutto chiaro?

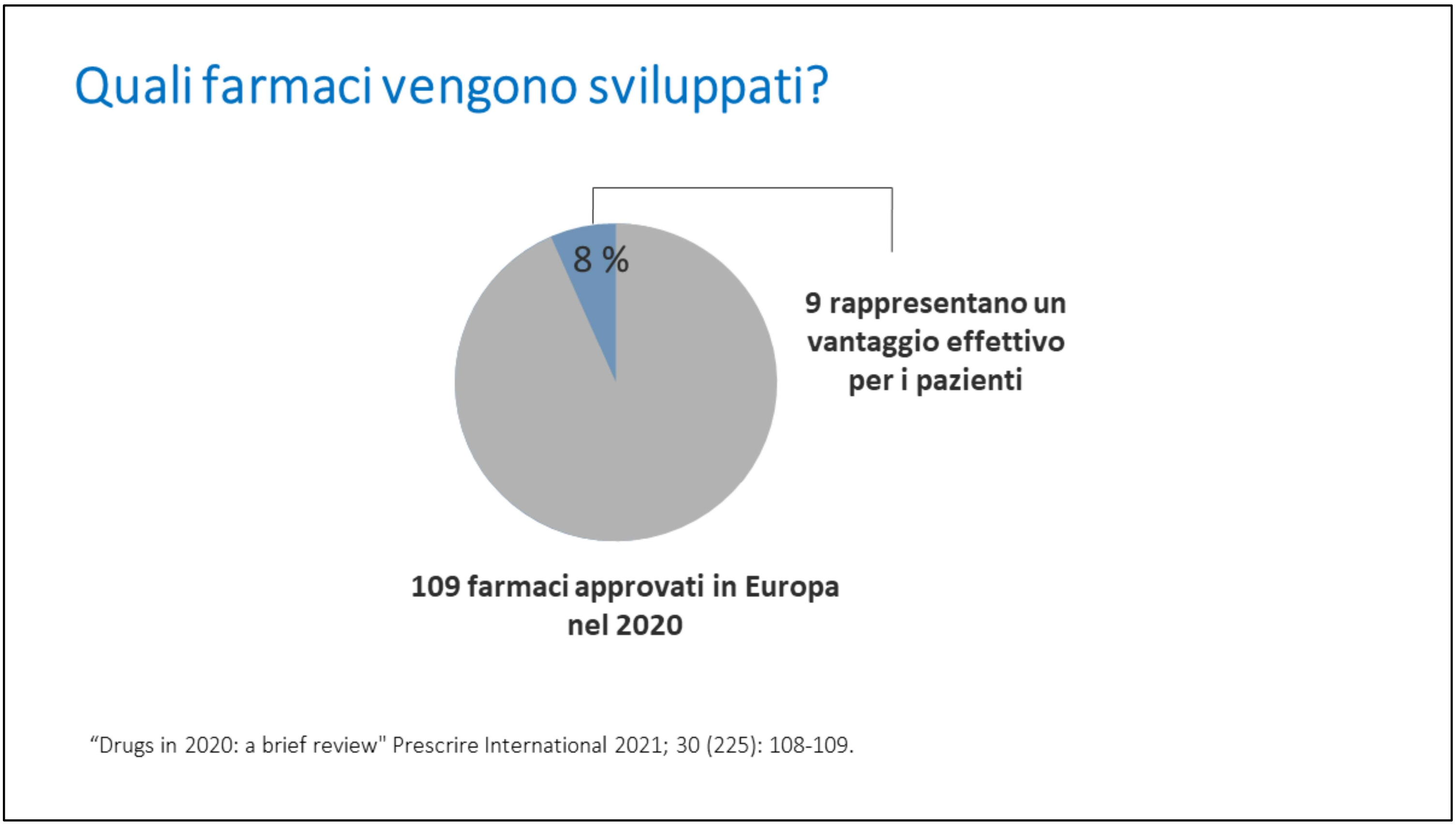

Domande:

- Avete domande? Commenti? Vi sembra tutto chiaro?
- Secondo voi, i nuovi farmaci sono sempre meglio di quelli già in uso?

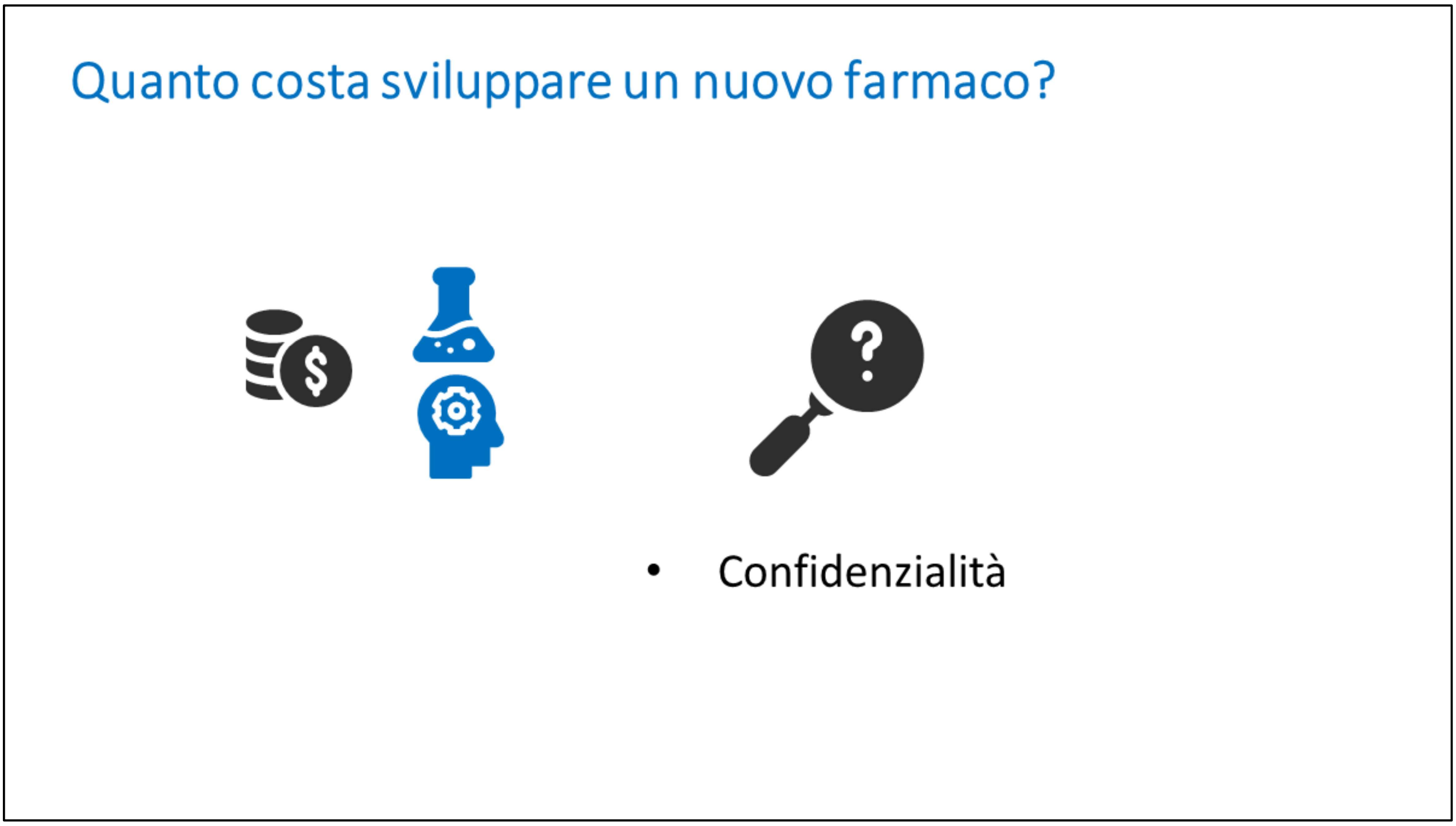

Domande:
Quali sono le vostre opinioni sulla confidenzialità di dati e informazioni sui costi necessari per lo sviluppo e immissione sul mercato dei farmaci? la confidenzialità dei costi va mantenuta/tutelata?

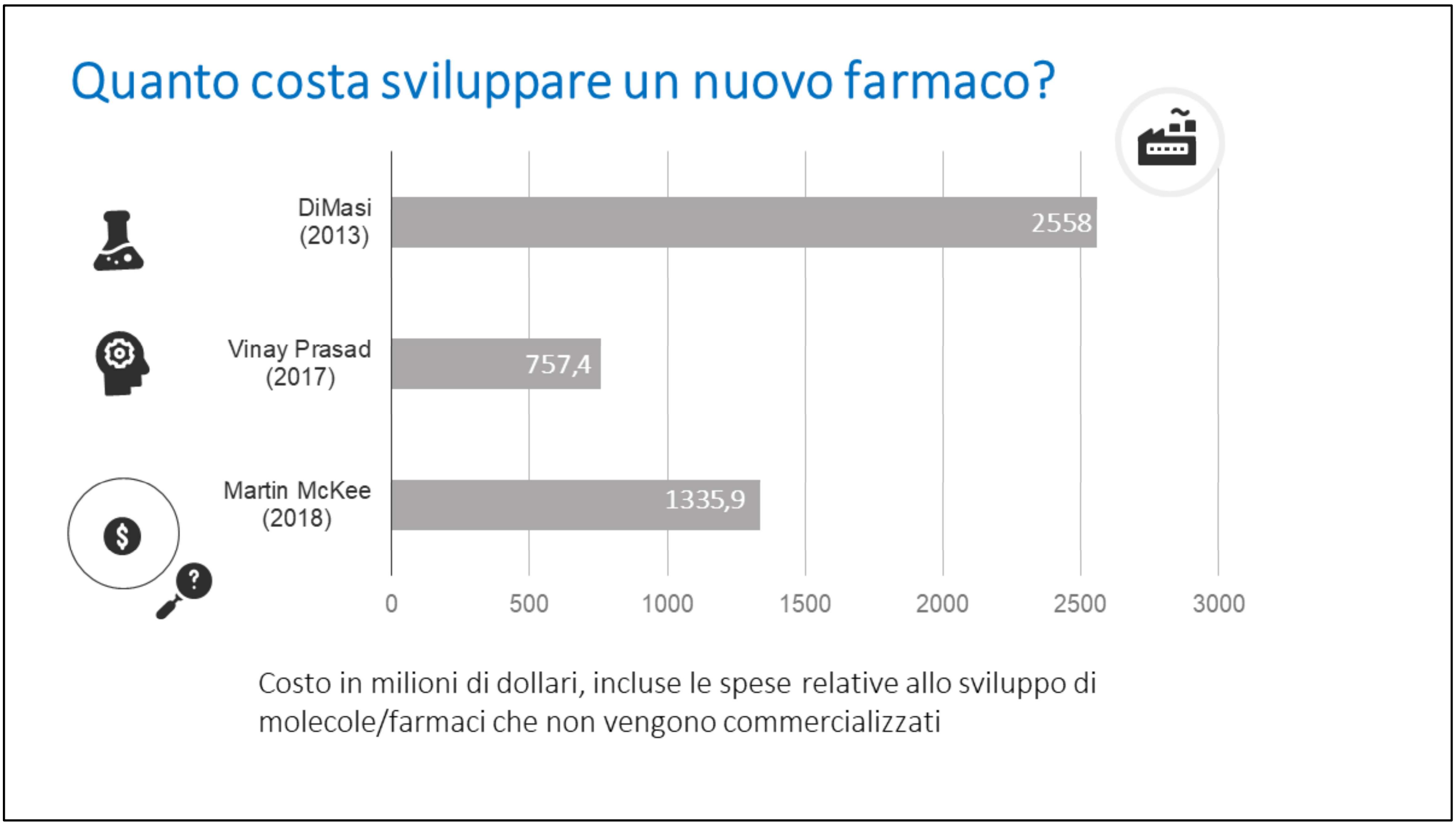

Domande:

Cosa ne pensate dei costi necessari per lo sviluppo e immissione sul mercato dei farmaci?

Rapporto tra costi di ricerca e sviluppo e ricavi dalle vendite ottenuti dalle aziende farmaceutiche

Esempio:
farmaci
antitumorali

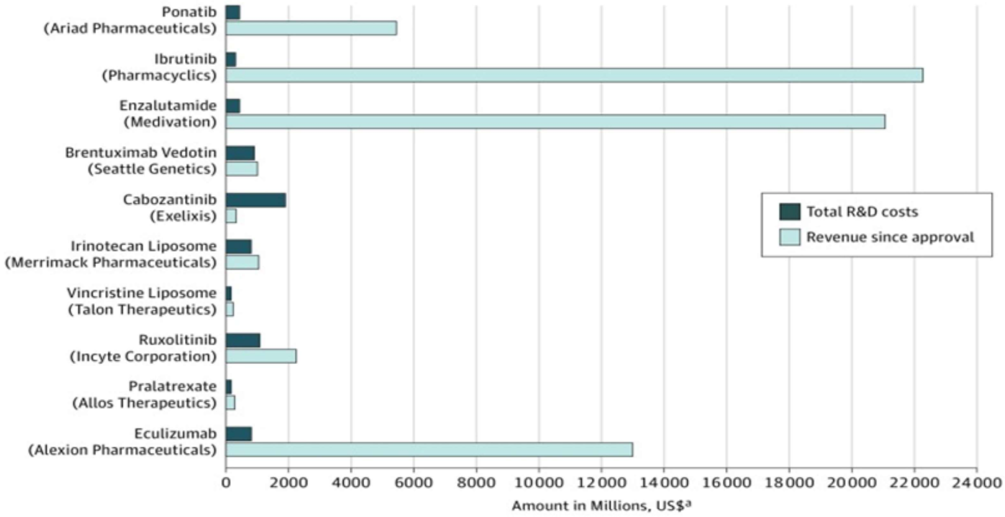

Prasad V, Mailankody S. Research and Development Spending to Bring a Single Cancer Drug to Market and Revenues After Approval. JAMA Intern Med. 2017;177(11):1569–1575

Domanda:
Cosa ne pensate dei ricavi delle aziende, rispetto ai costi di ricerca e sviluppo?

Finanziamento in ricerca e sviluppo di farmaci da parte delle aziende farmaceutiche e finanziamento in ricerca sanitaria (inclusi farmaci) da parte di enti pubblici

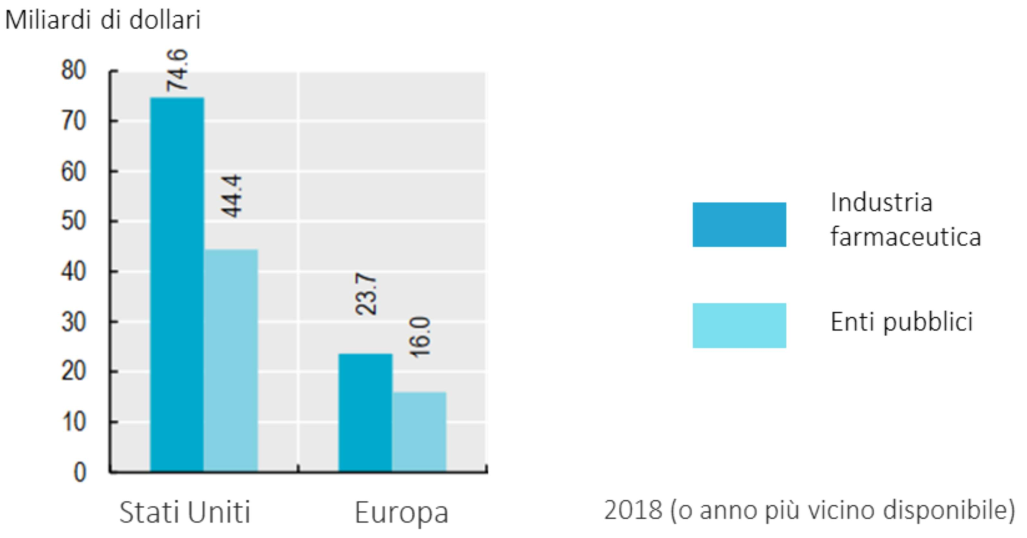

Tratto da: OECD (2021), "Business enterprise expenditure on pharmaceutical R&D and government budgets for health-related R&D, 2018 (or nearest year)", in *Health at a Glance 2021: OECD Indicators*, OECD Publishing, Paris, https://doi.org/10.1787/a2fd45e4-en.

Domanda

Cosa ne pensate del rapporto tra investimenti privati dell'industria farmaceutica e quelli pubblici nello sviluppo dei nuovi farmaci?

Semplificando...

- Il processo di ricerca e sviluppo di nuovi farmaci è strettamente **regolamentato**
- Una piccola parte di nuovi farmaci rappresenta un **vantaggio effettivo** per le persone con la malattia; molti **si aggiungono** a quelli già presenti sul mercato; di molti **non sappiamo** se portano vantaggi effettivi
- Le aziende farmaceutiche hanno **costi alti** per la ricerca e lo sviluppo di nuovi farmaci e hanno **ricavi dalle vendite** dei farmaci che spesso superano i costi, a volte di tanto
- I costi di ricerca e sviluppo per farmaco sono **confidenziali**
- Anche gli **enti pubblici finanziano** la ricerca e lo sviluppo di farmaci.

Parte 03

Come viene stabilito il prezzo di un farmaco

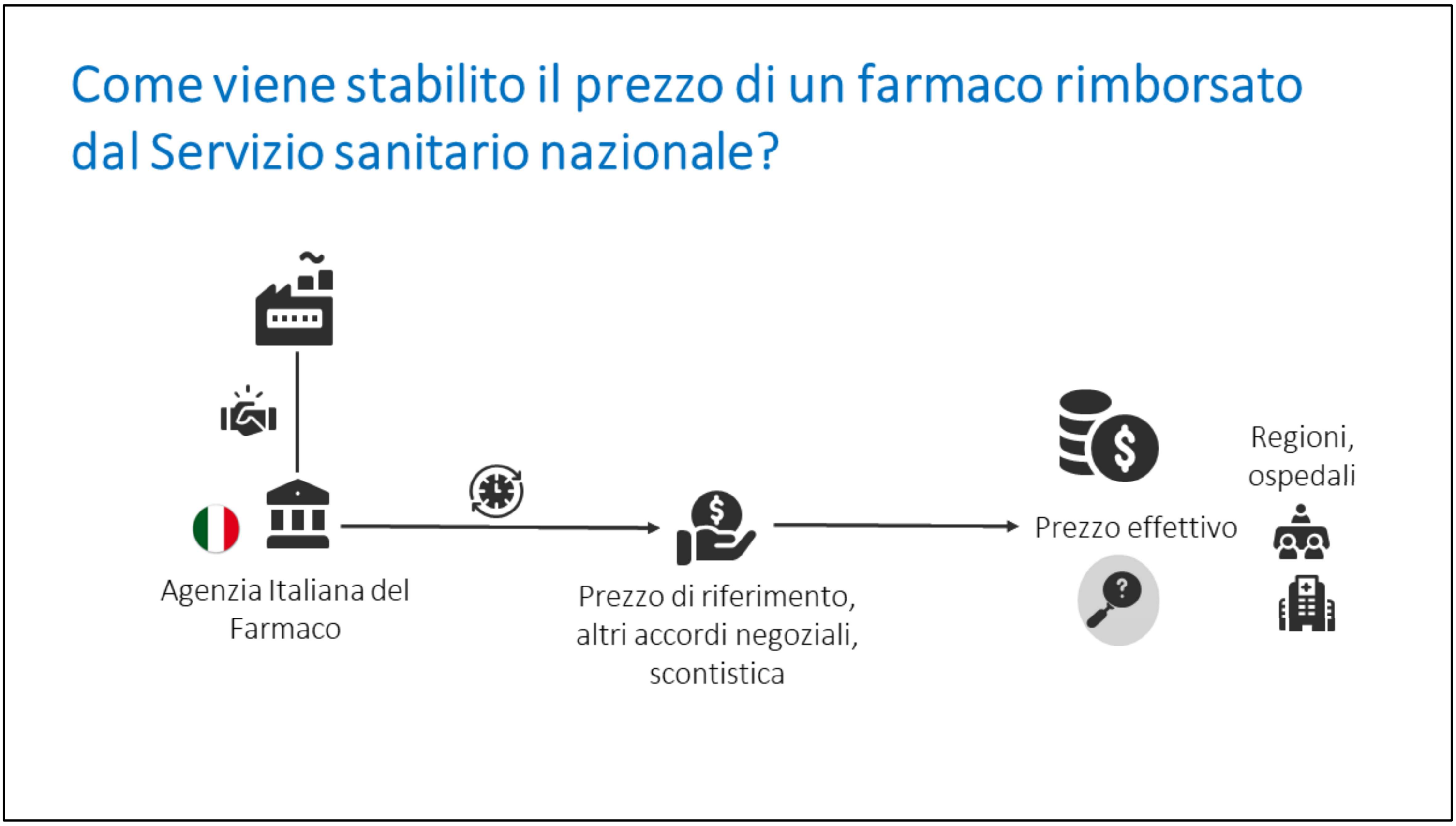

Domande:
Avete domande? Commenti? Vi sembra tutto chiaro?

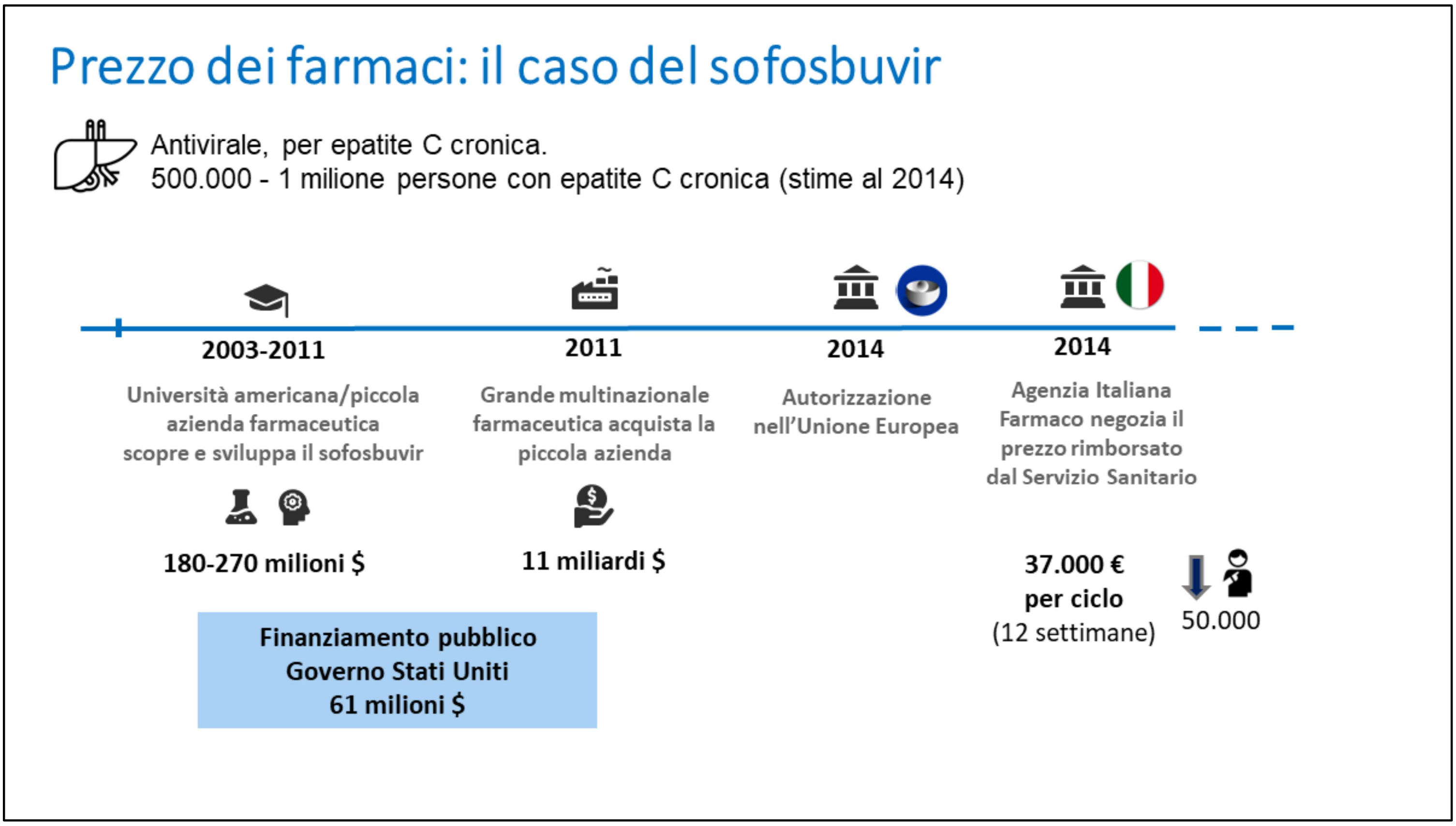

Domande:
Questo farmaco ha migliorato sostanzialmente la salute delle persone con Epatite C. Di fatto si può considerare un farmaco salvavita.

- Cosa pensate dei profitti che fa l'industria farmaceutica con la vendita di questo farmaco?
- Cosa ne pensate dei finanziamenti pubblici per la ricerca e lo sviluppo di nuovi farmaci?
- Come va considerato secondo voi il rischio finanziario (ricerca su molecole /farmaci che non arrivano in commercio) che le industrie farmaceutiche corrono nel processo di ricerca e sviluppo di nuovi farmaci?

Prezzo del sofosbuvir per 12 settimane di trattamento

Paese	Euro
Stati Uniti	61.700
Regno Unito	41.900
Germania	48.500
Francia	56.000
Egitto	660
Mozambico, Kenya, Myanmar	660
India	660
**Generico**	95-198
**Costo di produzione (stimato)**	50-99

Tratta da: «L'accesso ai nuovi farmaci negli altri Paesi» - Anna Maria Marata 10 luglio 2014, disponibile a: «Nuovi farmaci per l'epatite C: i materiali del convegno Iss» https://www.epicentro.iss.it/farmaci/HcvPresentazioni.

Domande

Cosa pensate della differenza di prezzi dello stesso farmaco? Avete considerazioni, o domande?

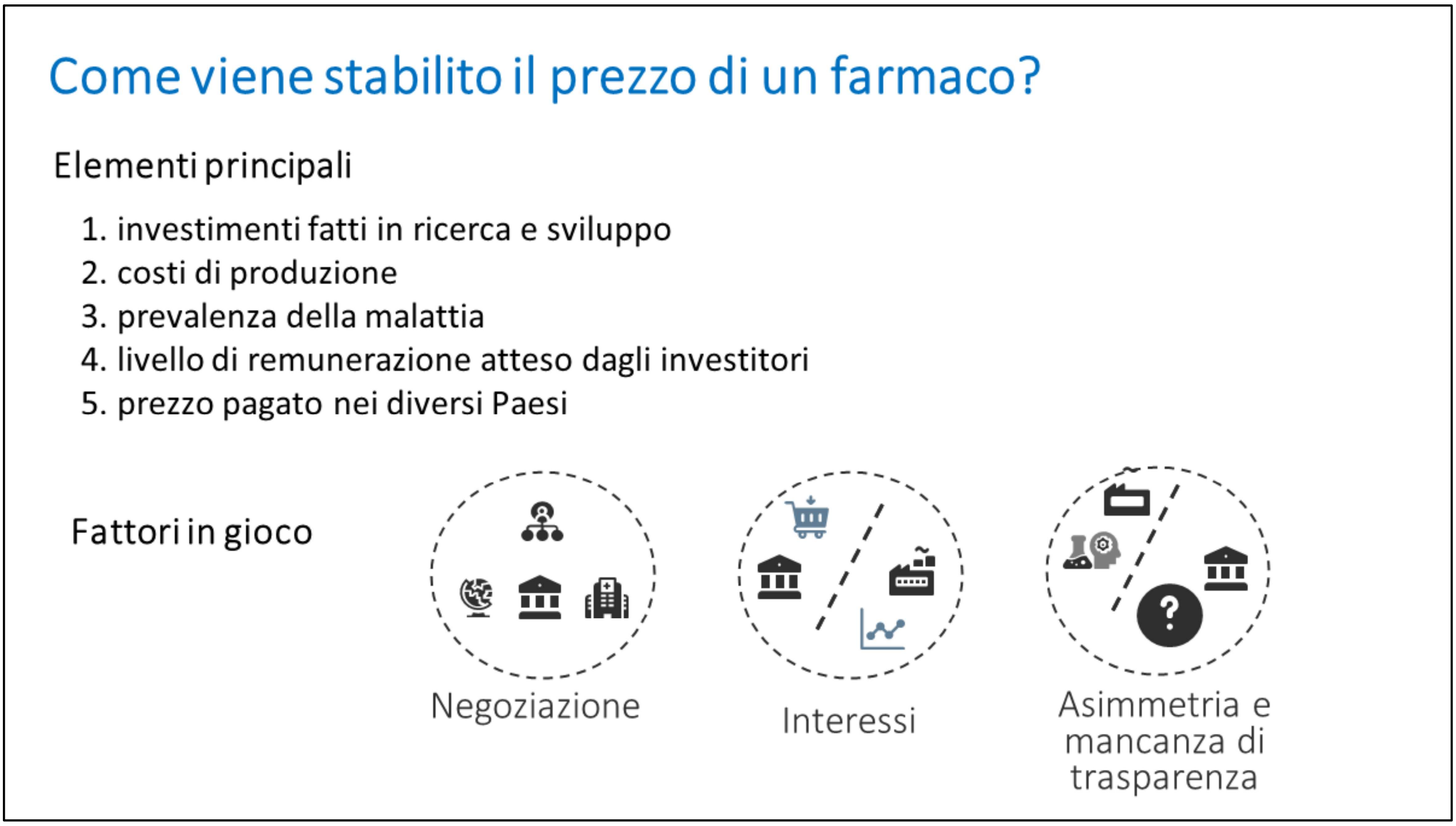

Domande:

- Cosa ne pensate di quello che è stato detto sulla mancanza di trasparenza nei processi di definizione del prezzo dei farmaci?
- Le autorità che stabiliscono il prezzo dei farmaci dovrebbero avere dalle industrie farmaceutiche informazioni dettagliate sui costi di ricerca e sviluppo, gli incentivi pubblici ricevuti, e il prezzo effettivo pagato da altri Paesi?
- Ritenete che questo possa influenzare la concorrenza tra industrie produttrici?

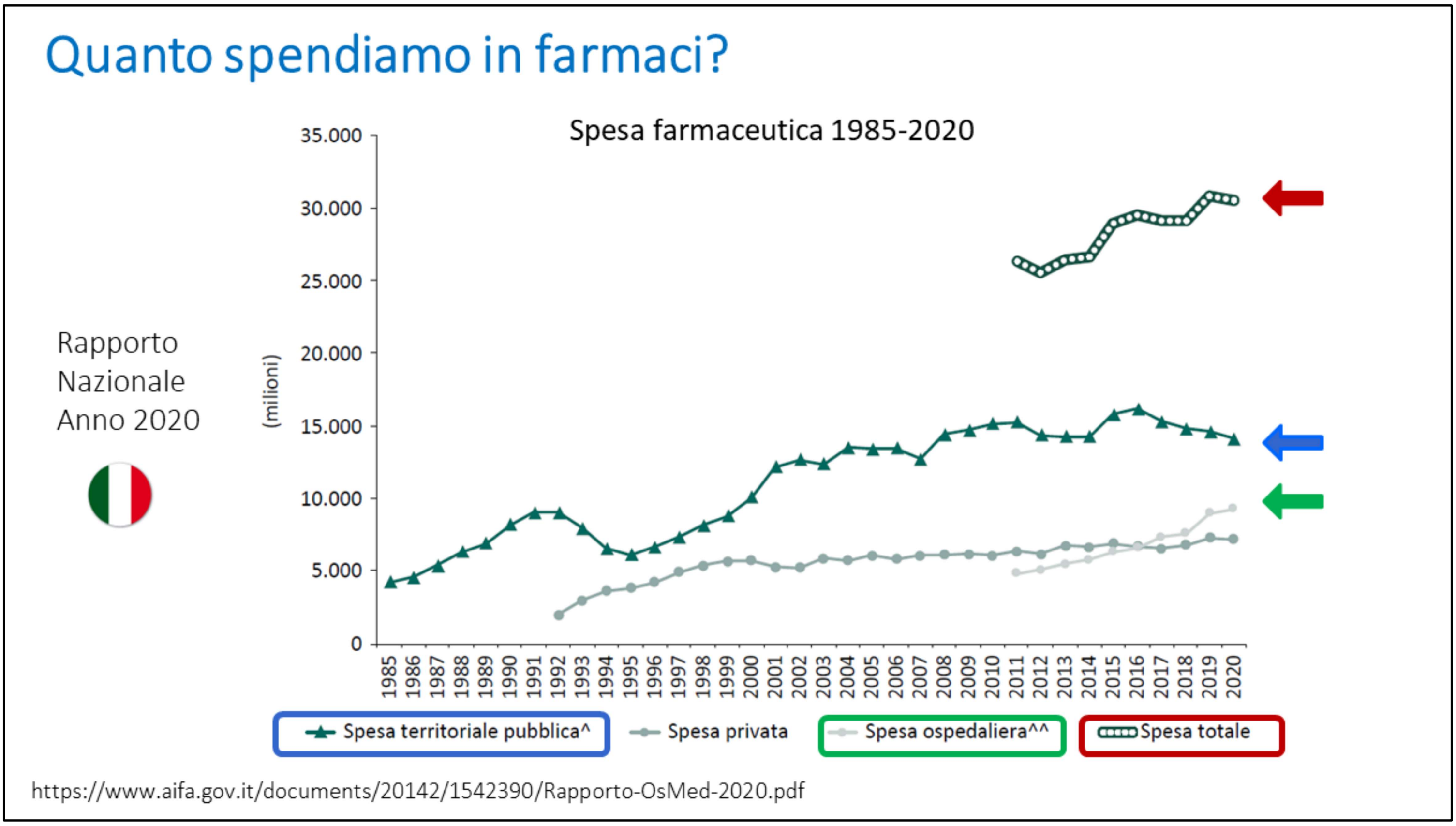

Domande:
Avete domande? Commenti?

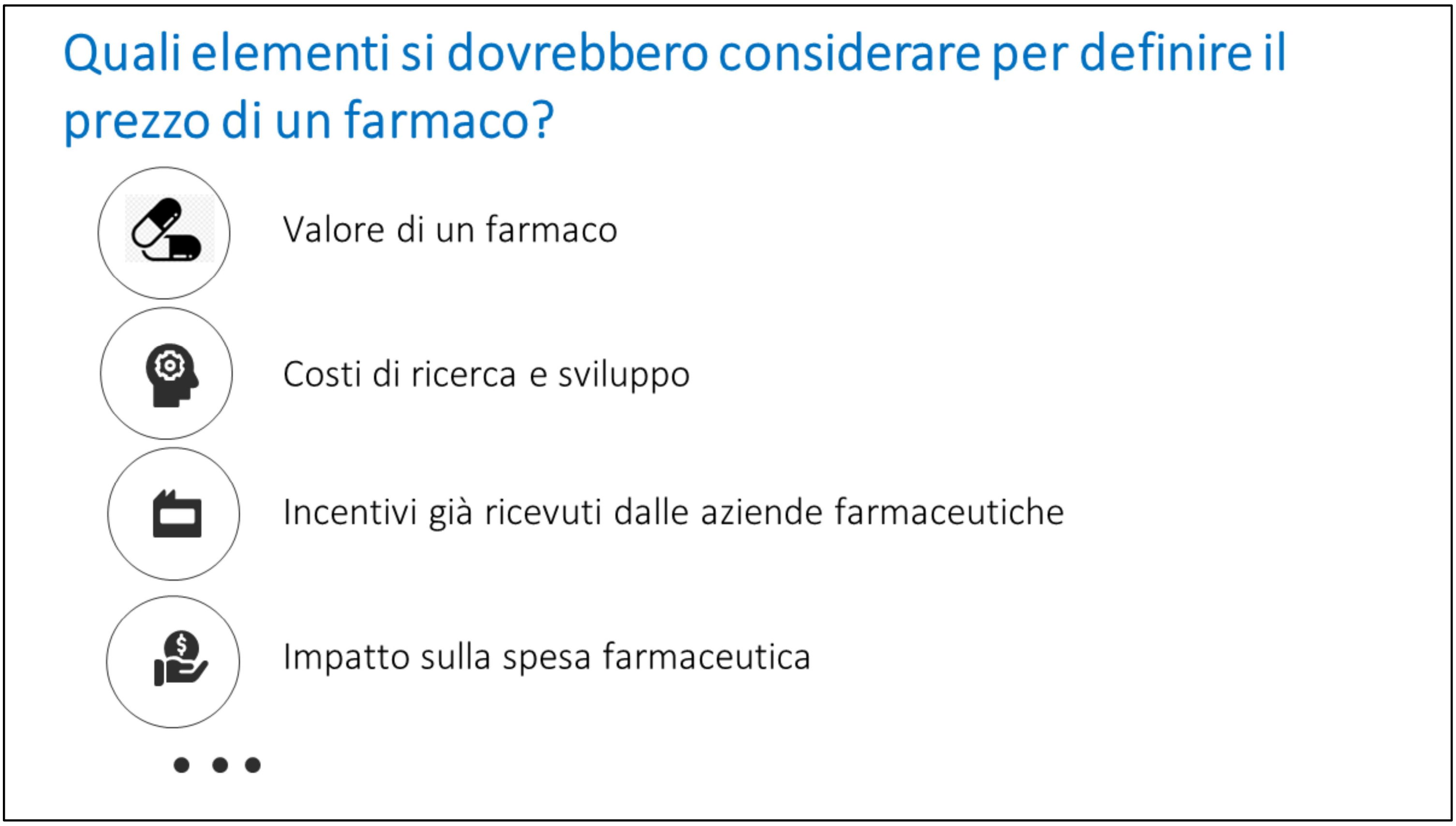

Domande:

- Andrebbero considerati altri fattori, aspetti..?
- Tra questi, quale considerate il più importante per definire il prezzo?
- Quale valore dovrebbe avere un *nuovo* farmaco? Quali benefici porta un nuovo farmaco alla società?

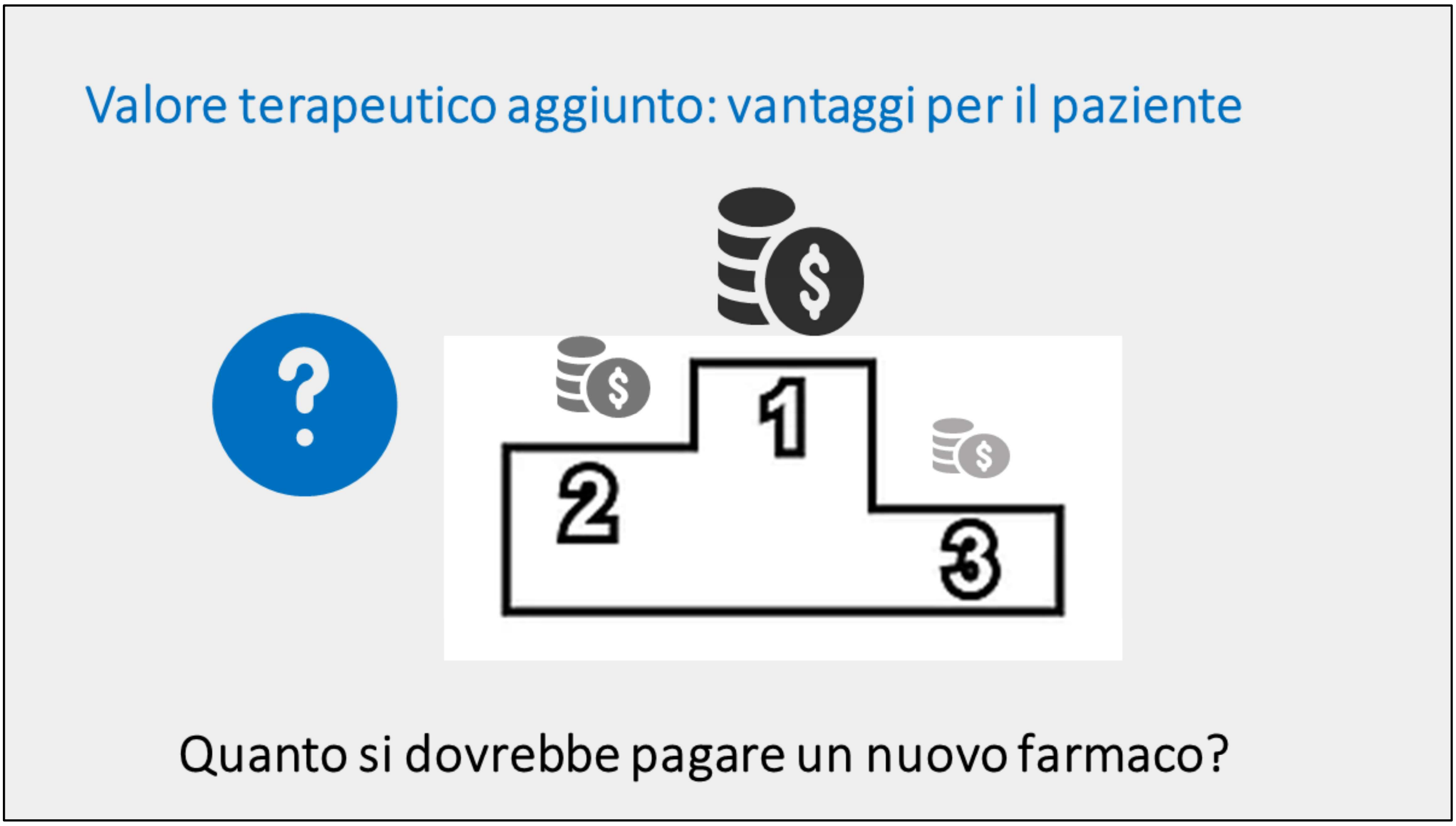

Domande:

- Nel definire il prezzo di un farmaco, va considerato – e come – il suo valore terapeutico aggiunto? I prezzi devono essere correlati a quanto un nuovo farmaco funziona? Vale a dire, pagare di più per quello che dà più vantaggi e di meno per quello che dà meno vantaggi?

Grazie per la partecipazione!
