# Supplementary material for: Citizens' views on prices of medicines reimbursed by the National Health Service: Findings from Italian online focus groups
Source: Health Expect. 2024 Mar 3;27(2):e14005. doi: 10.1111/hex.14005 (PMC10909621; doi:10.1111/hex.14005)
Supplement: Supplementary file 2 — Supporting information. [file HEX-27-e14005-s003.pdf]

Appendix 1b. Slide presentation and questions – English translation.

Slides presented during the meeting and main questions asked to participants.

The questions were adapted in each meeting based on the discussion.

The original version of the slides and questions can be found in Appendix 1a.

Citizens’ opinions and views on aspects related to the National Health Service and health

What we are going to talk about today in particular

The prices of medicines reimbursed by the National Health Service

Part 01

Opinions on medicines and the National Health Service

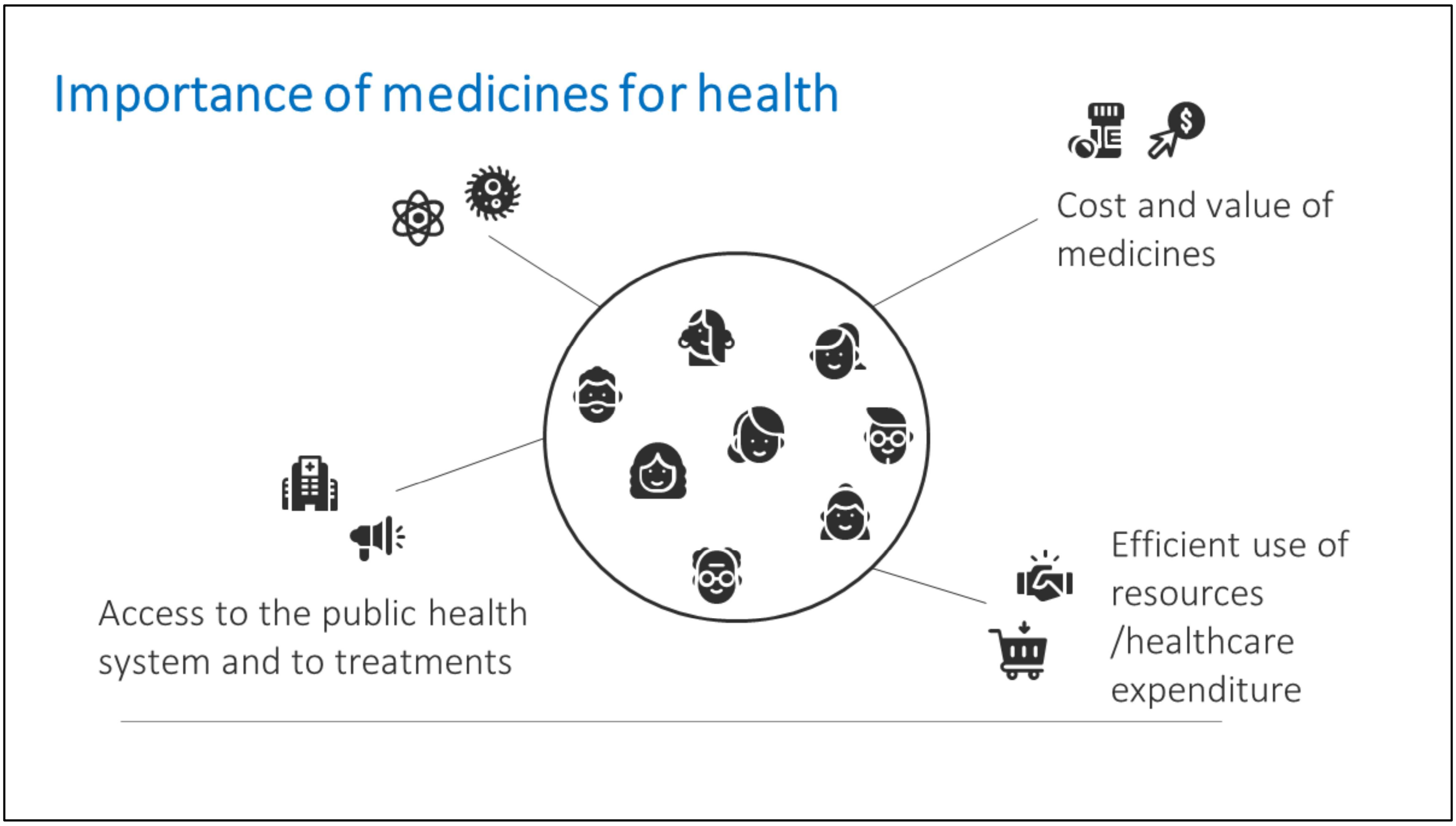

Questions

- In general terms, what value do you place on health?
- And to health care assistance?
- What value do medicines have in your health?
- Have you ever wondered what is the price of the medicines we use that are reimbursed by the public health service? (for example, diabetes medicines, or medicines used to prevent blood clots such as oral anticoagulants).
- How much do you think it costs to bring a new medicine to market? (order of magnitude: ....)
- Do you know what is the pathway to bring a new medicine to market?

02 Part

Development and cost of medicines

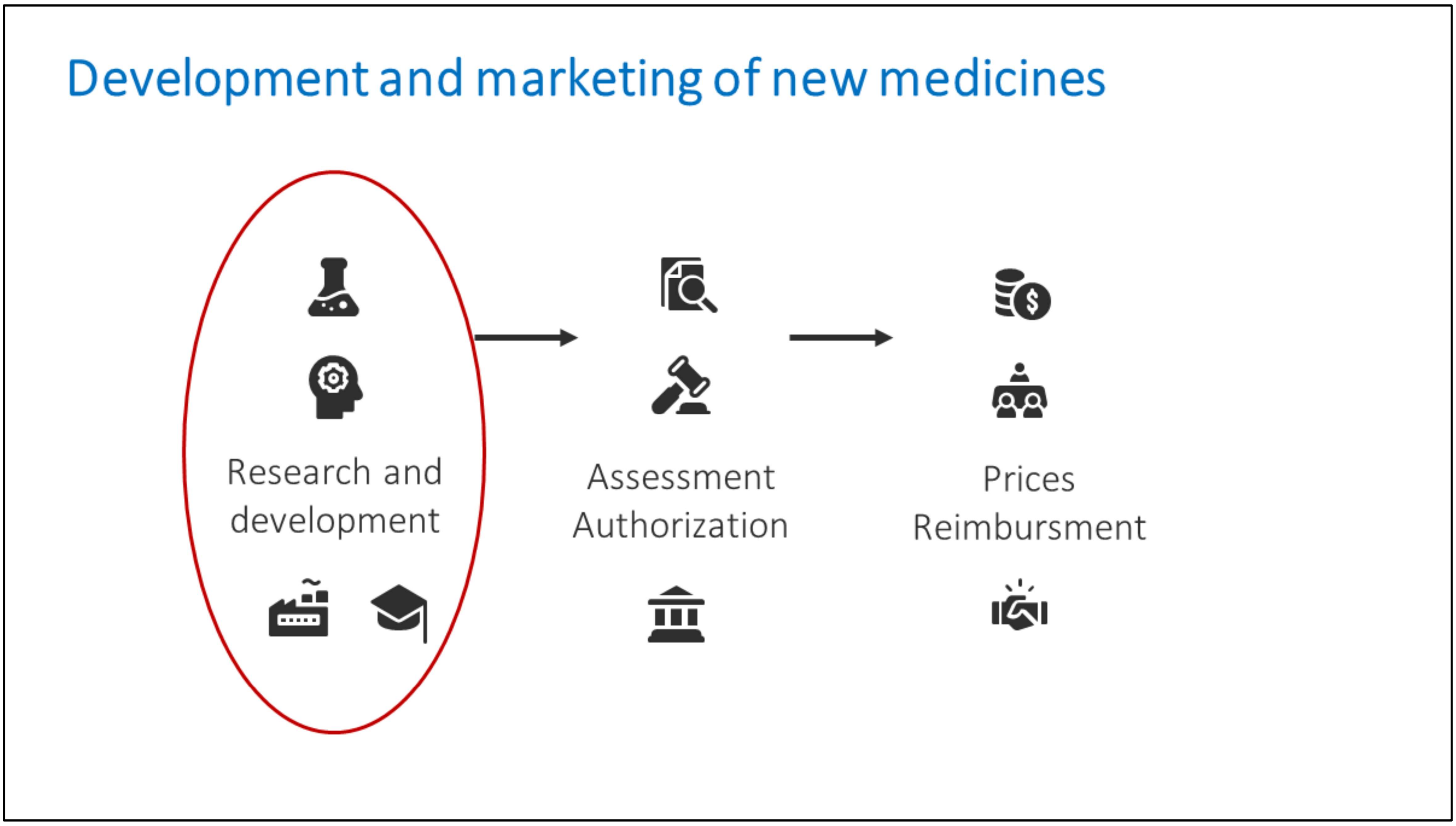

Question

- In your opinion, who funds the research and development of new medicines?

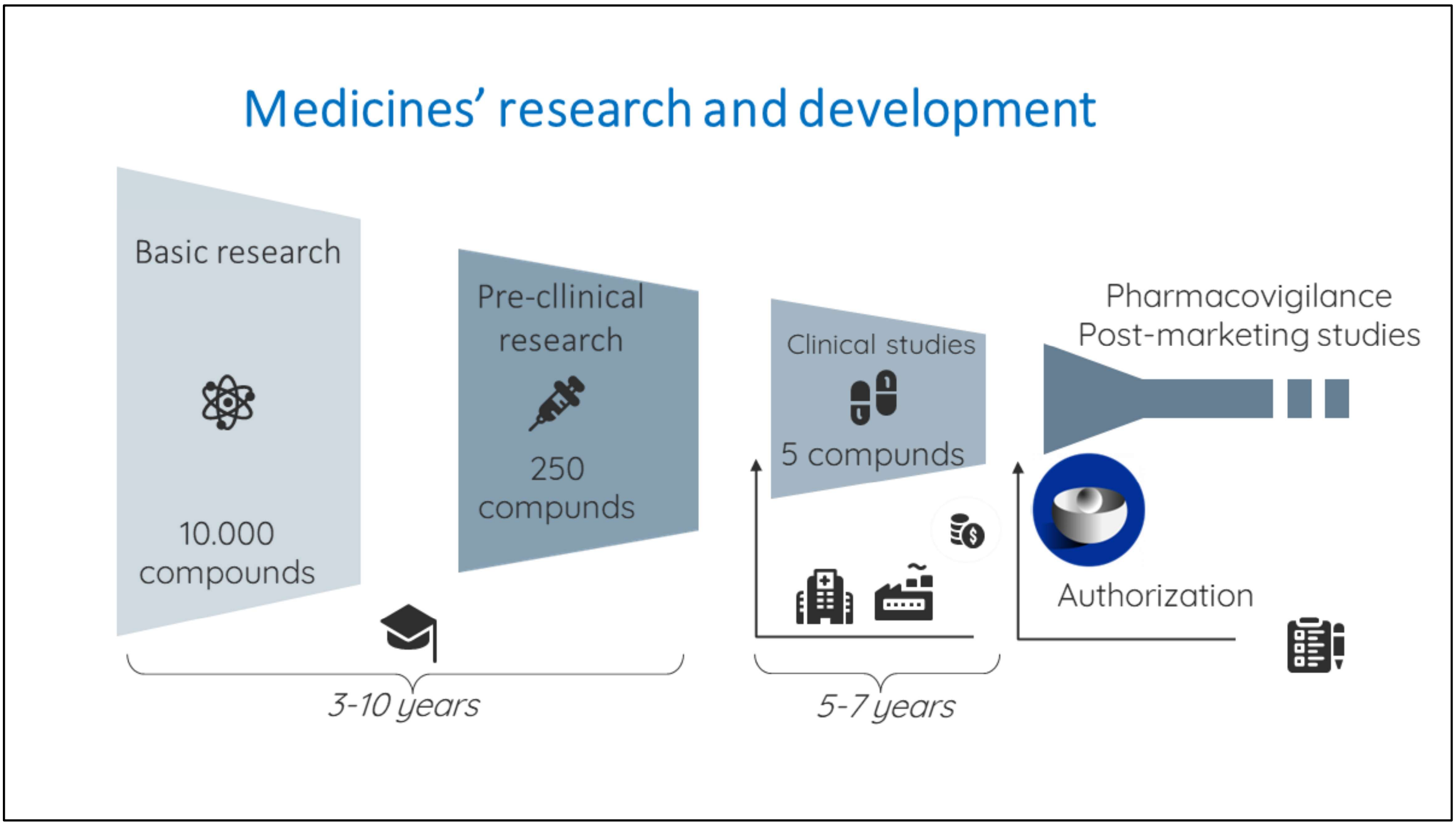

Questions

- Do you have any questions? Comments? Is everything clear?
- In your opinion, why/based on what is the decision to study or develop a new medicine...?
- In your opinion, are new medicines always better than those already in use?

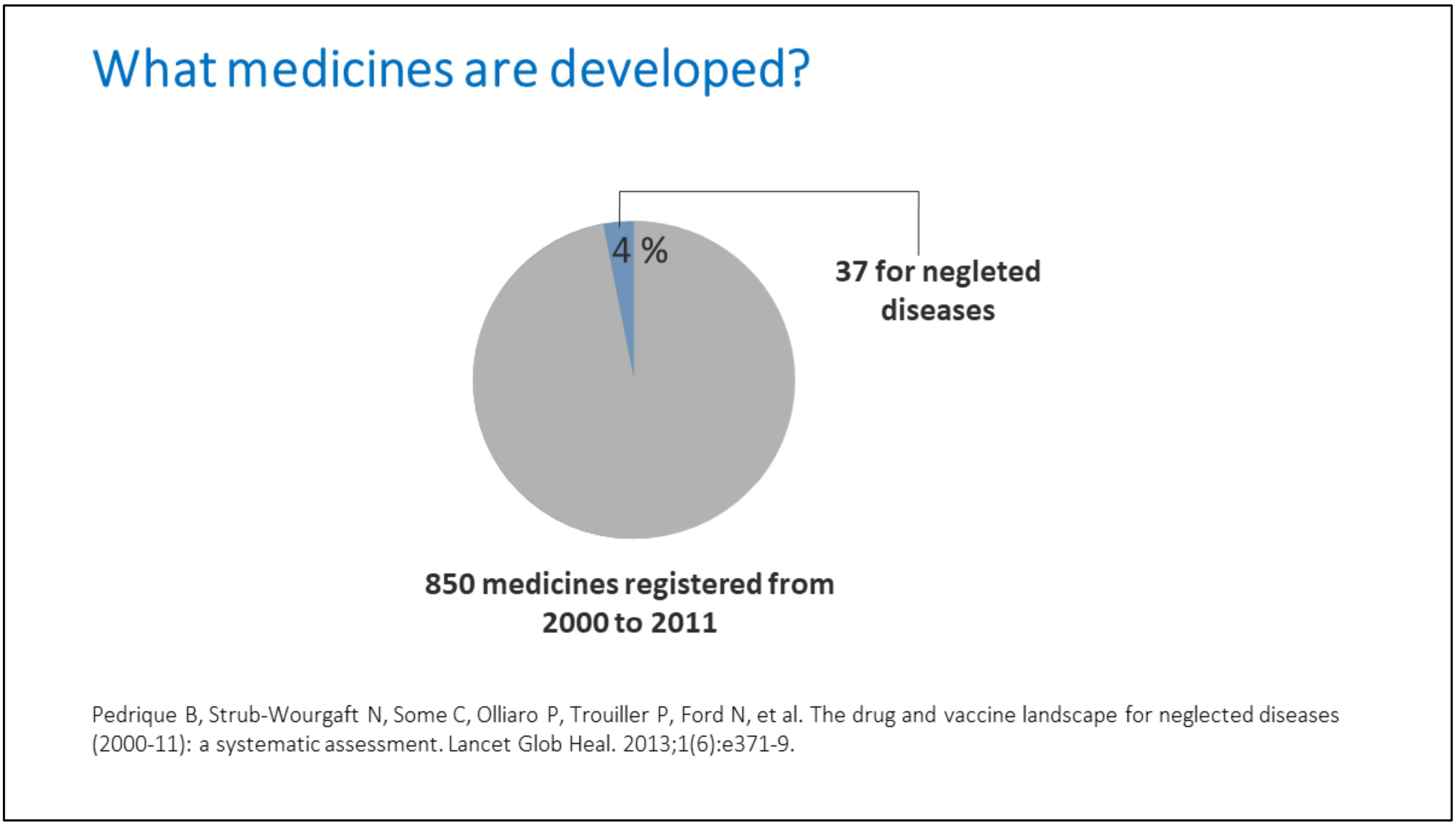

Questions

- Do you have any questions? Comments? Is everything clear?

What medicines are developed?

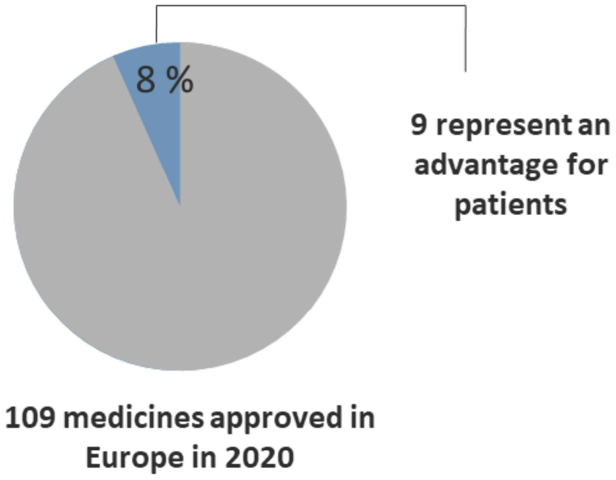

“Drugs in 2020: a brief review" Prescrire International 2021; 30 (225): 108-109.

Questions

- Do you have any questions? Comments? Is everything clear?
- In your opinion, are new medicines always better than those already in use?

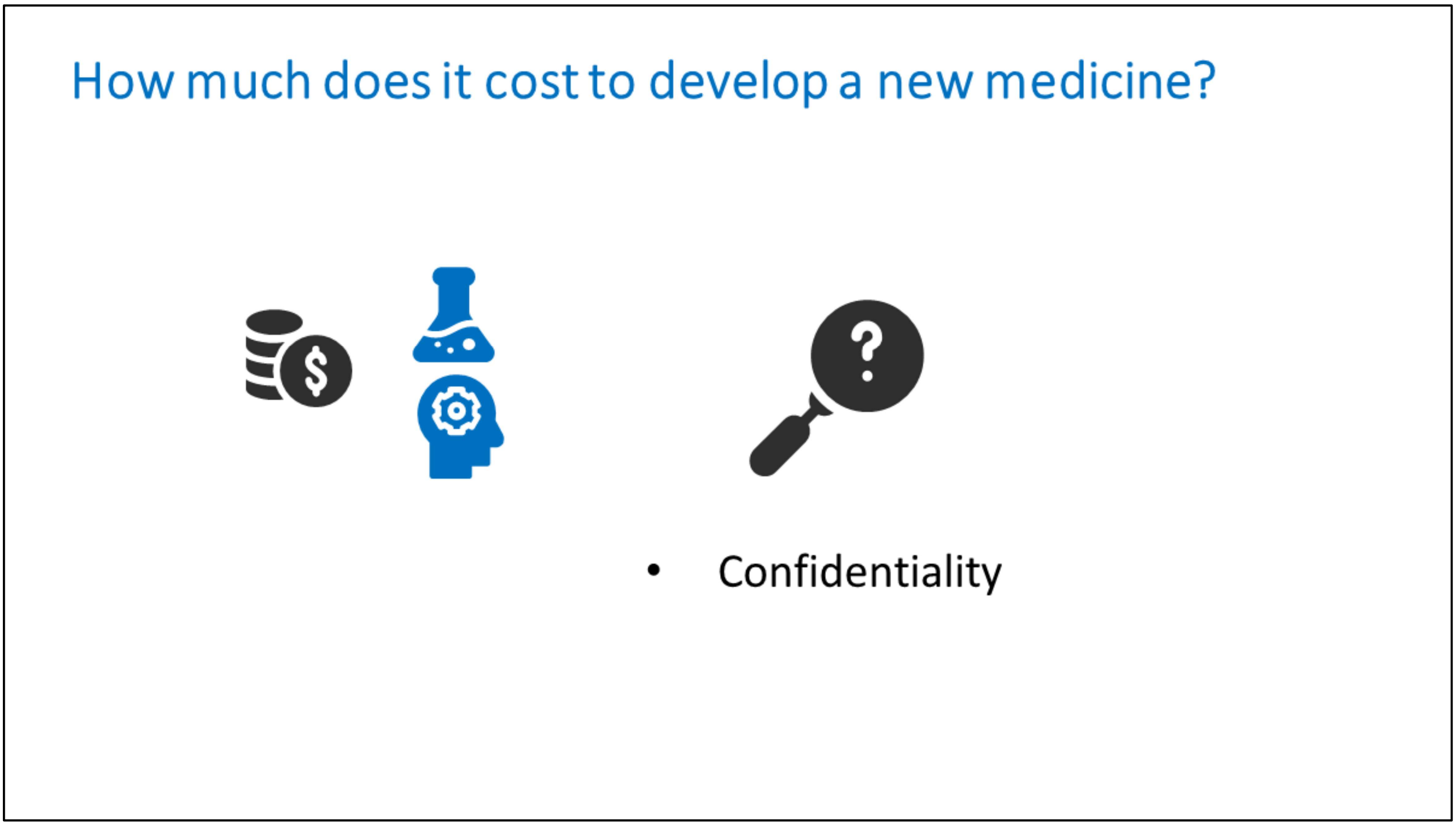

Questions

What are your views on the confidentiality of data and information on costs required for medicine development and marketing? Should cost confidentiality be maintained/protected?

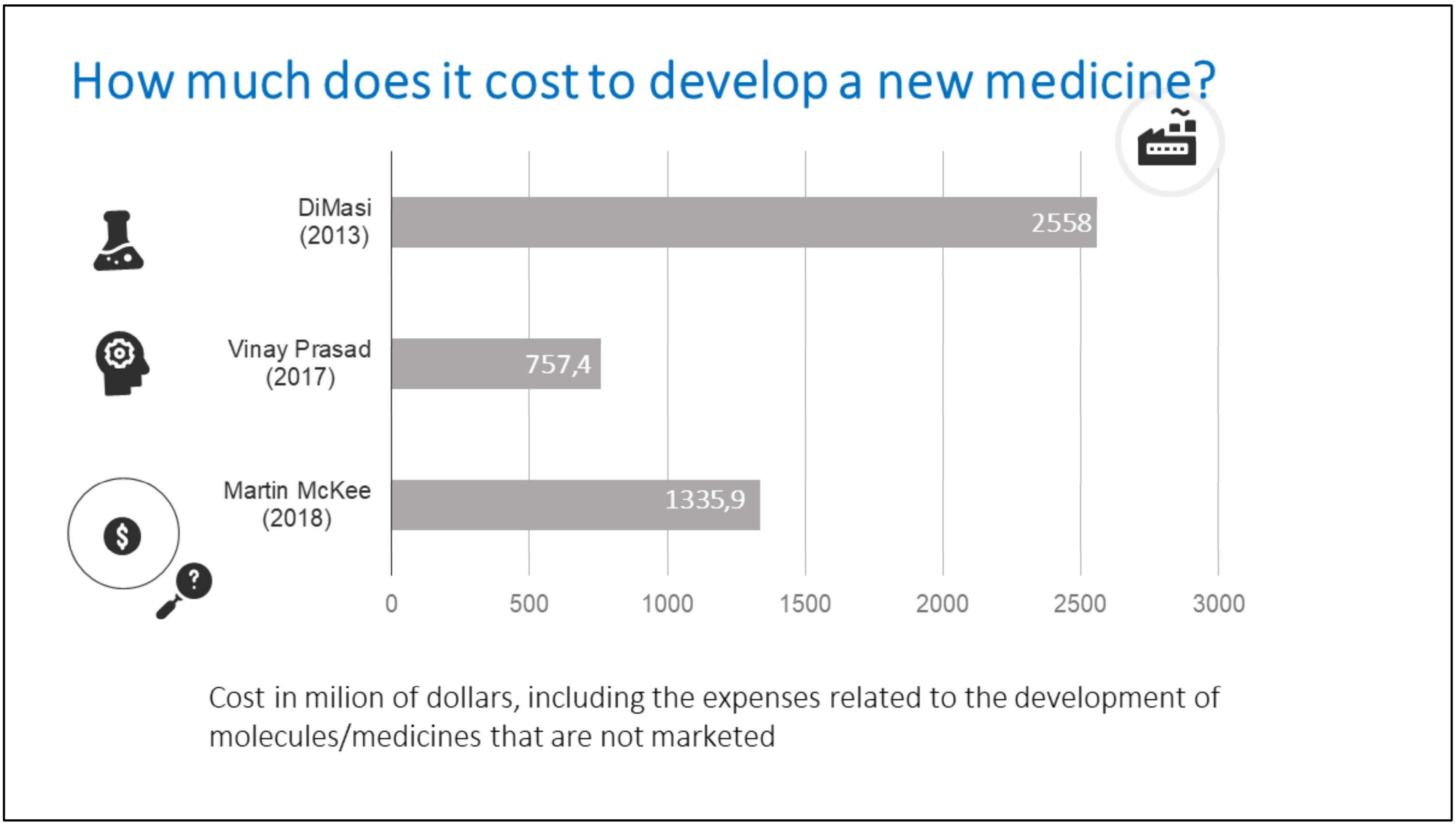

Question

What do you think about the costs of developing and bringing medicines to market?

Relation between research and development costs and sales revenues obtained by the pharmaceutical companies

Example: anticancer medicines

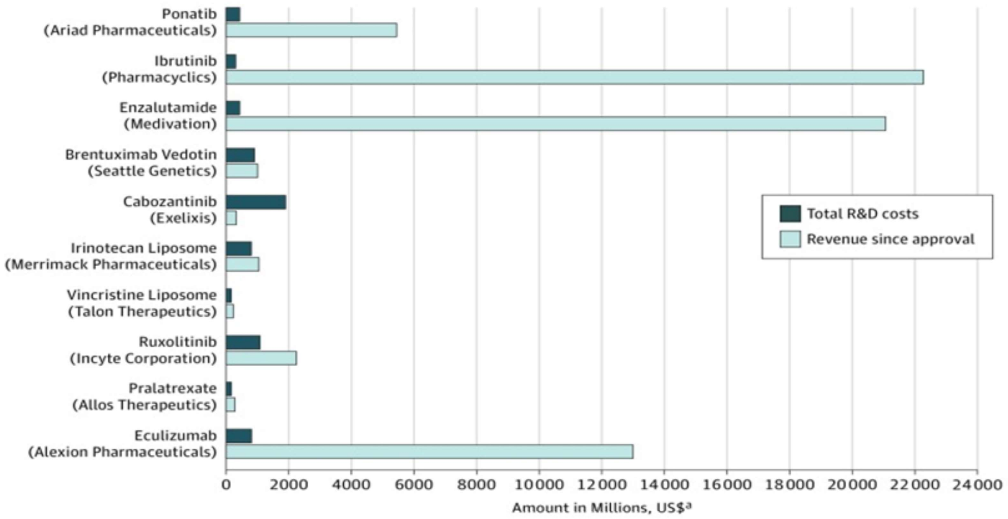

Prasad V, Mailankody S. Research and Development Spending to Bring a Single Cancer Drug to Market and Revenues After Approval. JAMA Intern Med. 2017;177(11):1569–1575

Question

What do you think about the revenues by pharmaceutical industries, compared to the research and development costs?

Funding in medicine research and development by pharmaceutical companies and funding in health research (including medicines) by public entities.

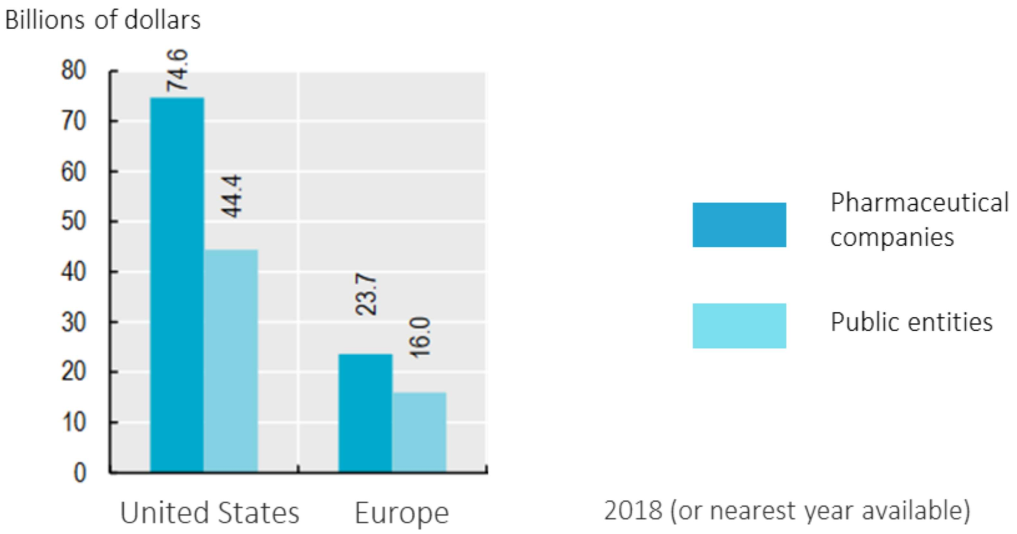

From: OECD (2021), "Business enterprise expenditure on pharmaceutical R&D and government budgets for health-related R&D, 2018 (or nearest year)", in *Health at a Glance 2021: OECD Indicators*, OECD Publishing, Paris, https://doi.org/10.1787/a2fd45e4-en.

Question

What do you think about the pharmaceutical industry investment and public investment in new medicine development?

Simplifying ...

- The process of research and development of new medicines is **strictly regulated**
- A small proportion of new medicines brings benefit to people with the disease; many **do not add** benefit compared to those on the market; for many **we do not know** whether they bring benefits
- Pharmaceutical companies have **high costs** for research and development of new medicines, and have **revenues** from medicine sales that **often exceed their costs**, sometimes by a lot.
- Research and development costs per medicine are **confidential**
- **Public entities** also **fund** medicines' research and development.

Part 03

How is the price of a medicine established?

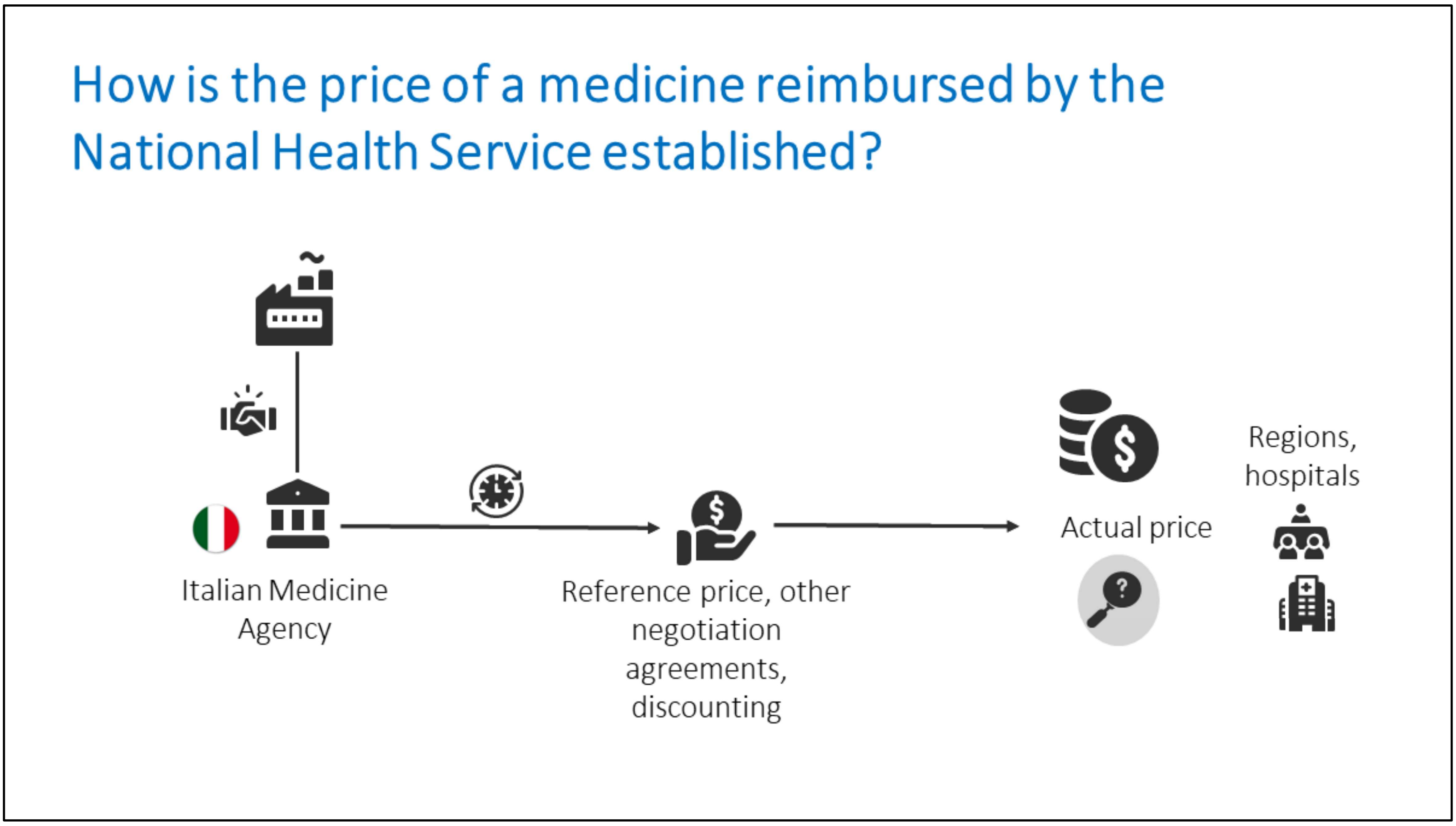

Questions

- Do you have any questions? Comments? Is everything clear?

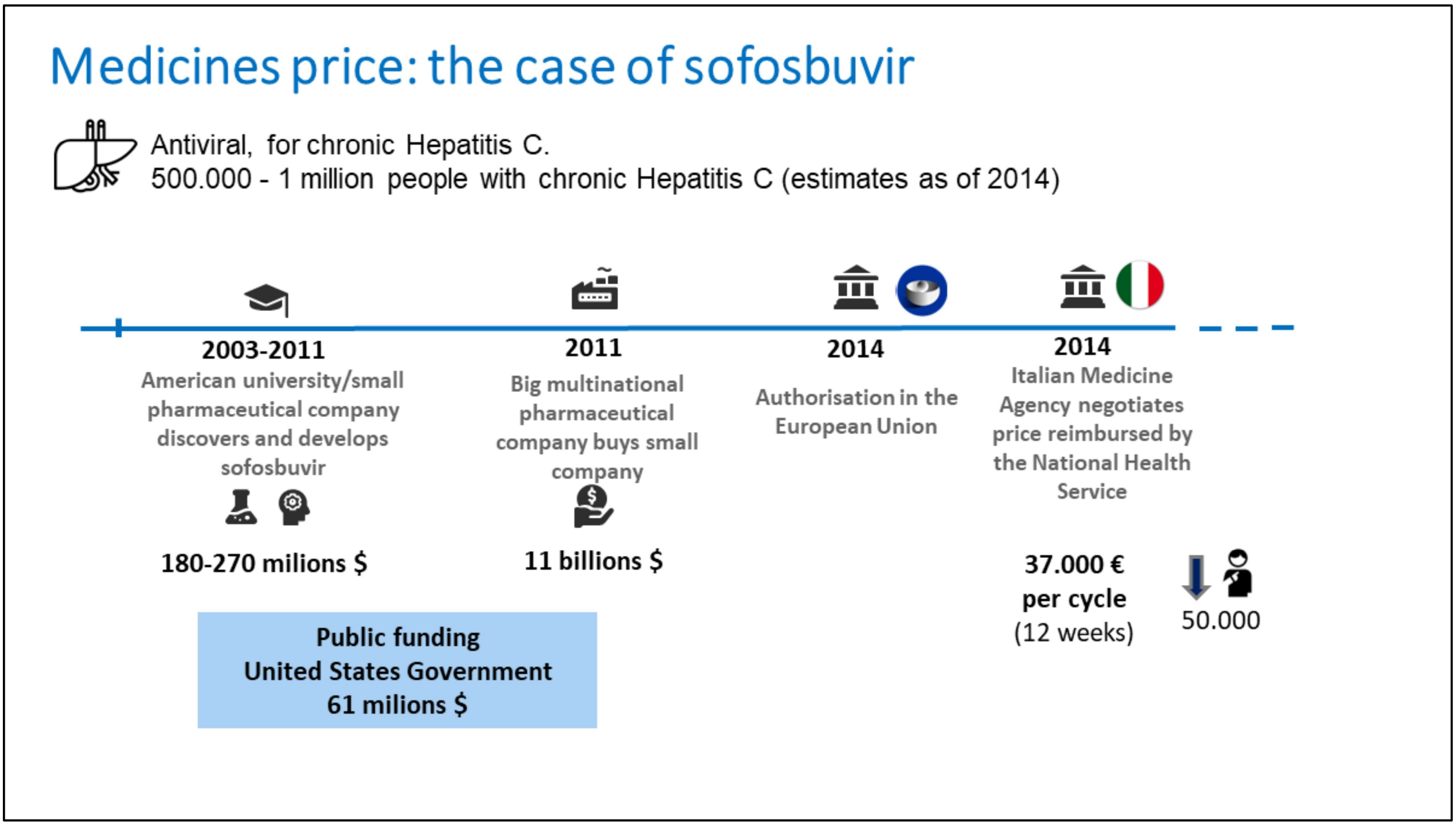

Questions

This medicine has substantially improved the health of people with Hepatitis C. It is in fact a life-saving medicine.

- What do you think about the profits the pharmaceutical industry makes from the sale of this medicine?
- What do you think about public funding for research and development of new medicines?
- How do you think the financial risk (research on molecules/medicines that fail) that pharmaceutical industries take in the process of new medicine research and development should be considered?

Price of sofosbuvir for 12 weeks of treatment

Country	Euros
United States	61.700
United Kingdom	41.900
Germany	48.500
France	56.000
Egypt	660
Mozambique, Kenya, Myanmar	660
India	660
**Generic**	95-198
**Production cost (estimated)**	50-99

From: «L'accesso ai nuovi farmaci negli altri Paesi» - Anna Maria Marata 10 luglio 2014, disponibile a: «Nuovi farmaci per l'epatite C: i materiali del convegno Iss» https://www.epicentro.iss.it/farmaci/HcvPresentazioni.

Questions

- What do you think about the price difference of the same medicine?
- Do you have any considerations, or questions?

How is the price of a medicine established?

Key elements

1. Investments made in research and development
2. Production costs
3. Prevalence of the disease
4. Level of remuneration expected by investors
5. Price paid in different Countries

Factors at stake

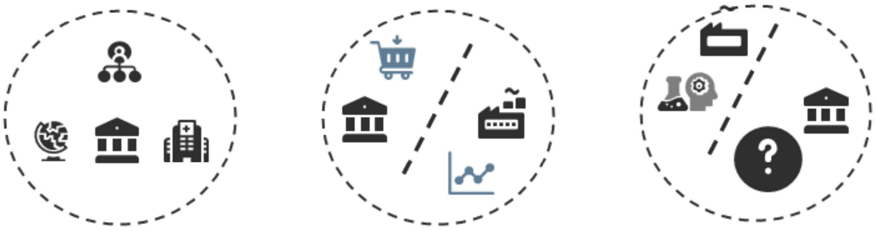

Negotiation | Interests | Asymmetry and lack of transparency

Questions

- What do you think about what has been said about the lack of transparency in medicine pricing processes?
- Should medicine pricing authorities have detailed information from pharmaceutical industries about research and development costs, public incentives received, and the actual price paid by Countries?
- Do you think this could affect competition among manufacturing industries?

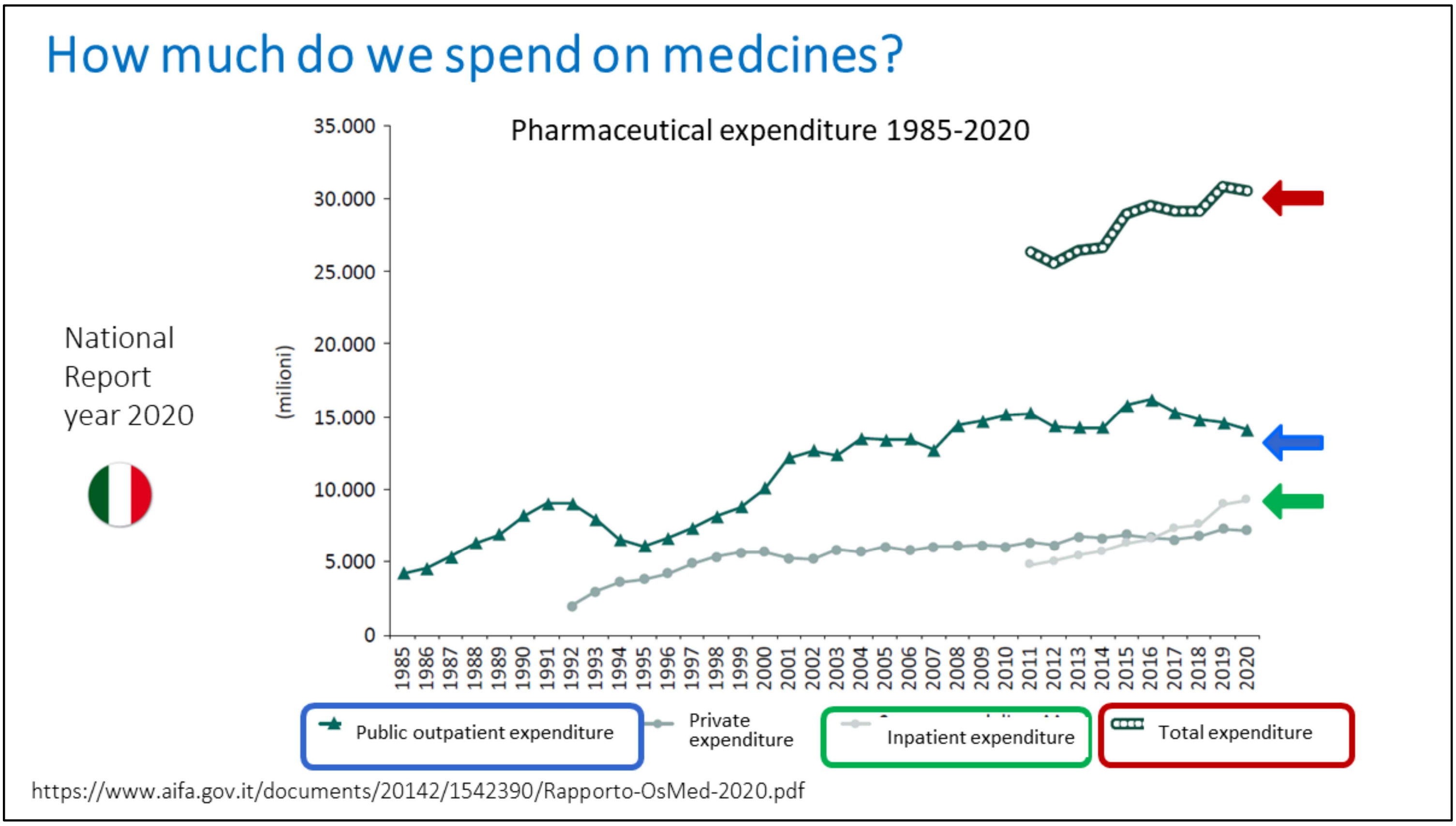

Questions

- Do you have any questions? Comments?

What elements should be considered when defining the price of a medicine?

- The value of a medicine
- Research and development costs
- Incentives already received by the pharmaceutical companies
- Impact on the pharmaceutical expenditure
- …

Questions

- Should other aspects be considered?
- Of these, which do you consider the most important in defining price?
- What value should a new medicine have?
- What benefits does a new medicine bring to society?

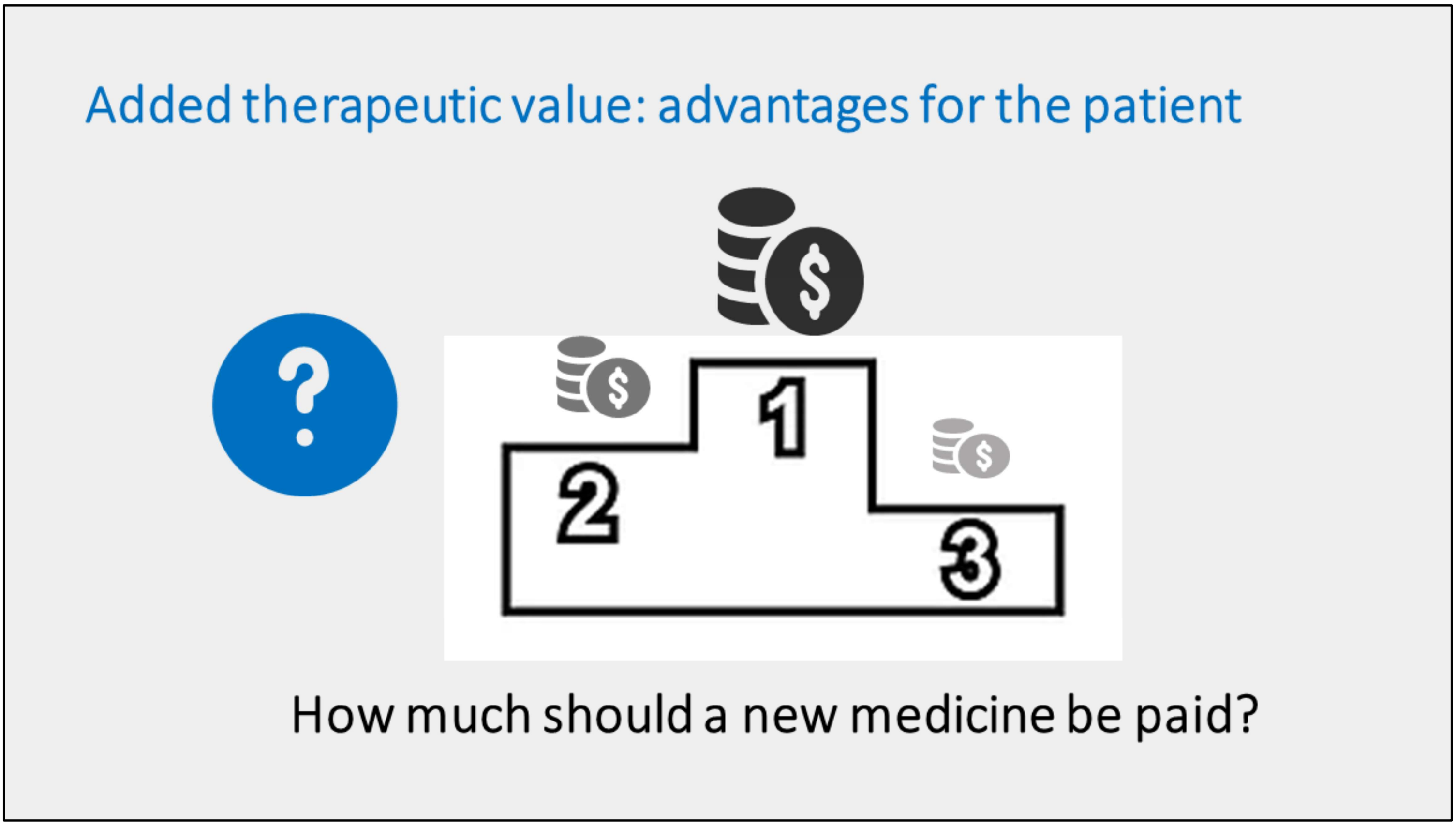

Questions

- When defining the price of a medicine, should its added therapeutic value be considered, and how?
- Should prices be related to how well a new medicine works? That is, pay more for what gives more benefit and less for what gives less benefit?

Thanks for your participation!
