# Supplementary material for: Citizens' views on prices of medicines reimbursed by the National Health Service: Findings from Italian online focus groups
Source: Health Expect. 2024 Mar 3;27(2):e14005. doi: 10.1111/hex.14005 (PMC10909621; doi:10.1111/hex.14005)
Supplement: Supplementary file 3 — Supporting information. [file HEX-27-e14005-s002.docx]

**Appendix 2.**

**Quotations from Italian focus groups, by main and sub-themes.**

**Favourable attitude to public health**

***Calling for a stronger public role with some concern from a few***

“Perhaps we have delegated too much to ... the pharmaceutical industry, and the State has delegated – maybe rightly, as the State, Italy in particular, is unable to carry out research with its own means, certainly”. *FG3*

***Wondering if public spending in R&D has a return***

“…non-repayable loan” *FG3*

**Calling for transparency**

***Asking for transparency, particularly on medicines’ prices***

“I think it (the cost) should be public, all public.” *FG3*

**Different perspectives on medicines’ value**

***Variable attitude to medicine use in daily life***

“I was very sick as a child, but now I take almost nothing. If I have to take something for toothache, I struggle to take it, in the sense that I rather try to put up with the pain”. *FG1*

“I am happy that there are medicines and vaccines”. *FG3*

“I am a fairly casual user of medicines ... if I have a problem, I want it to go immediately, so… like if I have a headache, I have to take something”. *FG2*

***Defining the value of new medicines is a complex issue***

“I was a little more optimistic, I usually did the “new-better medicine” equivalence .... “ *FG1*

“Me too (referring to a participant’s comment): new medicine = absolute innovation, that is to say a medicine you can trust ...” *FG1*

**Call for action to address high profits of pharmaceutical companies**

***Asking for a limit to pharmaceutical companies’ profits***

I expect that sooner or later we will arrive at a ceiling for these prices, because the national health system has to contribute to the health of every citizen, but not by speculating and enriching the pharmaceutical companies” *FG1*

“(…) my question...concerns precisely the presentation of medicines ... we all see…our family doctor, when the pharmaceutical representatives are lined up outside the room, waiting...” *FG3*

**Medicines’ pricing is a new issue for participants**

***Scant knowledge on R&D and on the negotiations of medicines’ prices***

“I think that ….they should go to the less explored fields, right? For diseases that may not be diagnosed or for which the therapeutic approach is difficult …rare diseases ... when there are such obvious problems with which the local health service, the national ... is unable to cope, probably there is the need ... I think, for more research and therefore for in-depth analysis”. *FG1*

“I think it is mainly on the basis of the impact, perhaps on the population? Maybe a certain range? That might be an incidence of – I don't know – 1 in 10,000… and then I don't know, on the basis of the situation or how it develops, that is, how much the effect might be on mortality. How much it can ... affect a certain area, a certain population, a certain number, that's it”. *FG4*
